# Supplementary material for: Novel South African Rare Actinomycete Kribbella speibonae Strain SK5: A Prolific Producer of Hydroxamate Siderophores including New Dehydroxylated Congeners
Source: Molecules. 2020 Jun 29;25(13):2979. doi: 10.3390/molecules25132979 (PMC7411998; doi:10.3390/molecules25132979)
Supplement: Supplementary file 1 [file molecules-25-02979-s001.pdf]

Supplementary Material

Article

Novel South African Rare Actinomycete *Kribbella speibonae* strain SK5: A Prolific Producer of Hydroxamate Siderophores including New Dehydroxylated Congeners

Kojo Sekyi Acquah[1], Denzil R. Beukes[2], Digby F. Warner[3,4,5], Paul R. Meyers[6], Suthananda N. Sunassee[1], Fleurdeliz Maglangit[7,8], Hai Deng[8], Marcel Jaspars[8], David W. Gammon[1*]

1 Department of Chemistry, University of Cape Town, Rondebosch 7701, South Africa; ACQKOJ001@myuct.ac.za, snsunassee@gmail.com, david.gammon@uct.ac.za

2 School of Pharmacy, University of the Western Cape, Bellville 7535, South Africa; dbeukes@uwc.ac.za

3 SAMRC/NHLS/UCT Molecular Mycobacteriology Research Unit & DST/NRF Centre of Excellence for Biomedical TB Research, Department of Pathology, Faculty of Health Sciences, University of Cape Town, Observatory 7925, South Africa; digby.warner@uct.ac.za

4 Institute of Infectious Disease and Molecular Medicine, Faculty of Health Sciences, University of Cape Town, Observatory 7925, South Africa; digby.warner@uct.ac.za

5 Wellcome Centre for Infectious Diseases Research in Africa, University of Cape Town, Rondebosch 7701, South Africa; digby.warner@uct.ac.za

6 Department of Molecular and Cell Biology, University of Cape Town, Rondebosch 7701, South Africa; paul.meyers@uct.ac.za

7 College of Science, University of the Philippines Cebu, Lahug, Cebu City 6000, Philippines; 01fm16@abdn.ac.uk

8 Marine Biodiscovery Centre, Department of Chemistry, University of Aberdeen, Old Aberdeen, AB24 3UE, Scotland, UK; m.jaspars@abdn.ac.uk

List of Figures

Figure S1. MS window of HPLC-DAD/HRESIMS profiles of MeOH (top), EtOAC (middle) and CH_2Cl_2 (bottom) extracts of the fermentation broth of *Kribbella speibonae* strain SK5 cultured in ISP2 liquid medium. 4
Figure S2. GNPS molecular network of the methanol (red nodes), ethyl acetate (blue nodes) and dichloromethane (green nodes) extracts of the fermentation broth of *K. speibonae* strain SK5. The molecular network contains 13 families with the highlighted families (desferrioxamines, ferrioxamines and diketopiperazines) containing some annotated nodes. 5
Figure S3. HR-ESI-MS spectrum of speibonoxamine (**1**) 6
Figure S4. ^{1}H-NMR spectrum of speibonoxamine (**1**) in DMSO-d6 at 600 MHz. 7
Figure S5. ^{13}C-NMR spectrum of speibonoxamine (**1**) in DMSO-d6 at 600 MHz. 8
Figure S6. Multiplicity edited HSQC NMR spectrum of speibonoxamine (**1**) in DMSO-d6 at 600 MHz. 9
Figure S7. ^{1}H-^{1}H COSY spectrum of speibonoxamine (**1**) in DMSO-d6 at 600 MHz. 10
Figure S8. HMBC NMR spectrum of speibonoxamine (**1**) in DMSO-d6 at 600 MHz. 11
Figure S9. HR-ESI-MS spectrum of desoxy-desferrioxmaine D_1 (**2**) 12
Figure S10. ^{1}H-NMR spectrum of desoxy-desferrioxmaine D_1 (**2**) in DMSO-d6 at 600 MHz. 13
Figure S11. ^{13}C-NMR spectrum of desoxy-desferrioxmaine D_1 (**2**) in DMSO-d6 at 600 MHz. 14
Figure S12. Multiplicity edited HSQC NMR spectrum of desoxy-desferrioxmaine D_1 (**2**) in DMSO-d6 at 600 MHz. 15
Figure S13. ^{1}H-^{1}H COSY spectrum of desoxy-desferrioxmaine D_1 (**2**) in DMSO-d6 at 600 MHz. 16
Figure S14. HMBC NMR spectrum of desoxy-desferrioxmaine D_1 (**2**) in DMSO-d6 at 600 MHz. 17
Figure S15. HR-ESI-MS spectrum of desferrioxamine D_1 (**3**) 18
Figure S16. ^{1}H-NMR spectrum of desferrioxamine D_1 (**3**) in DMSO-d6 at 600 MHz. 18
Figure S17. HR-ESI-MS spectrum of desferrioxamine B (**4**) 19
Figure S18. ^{1}H-NMR spectrum of desferrioxamine B (**4**) in DMSO-d6 at 600 MHz. 19
Figure S19. HR-ESI-MS spectrum of desoxynocardamine (**5**) 20
Figure S20. ^{1}H-NMR spectrum of desoxynocardamine (**5**) in DMSO-d6 at 600 MHz. 20
Figure S21. HR-ESI-MS spectrum of nocardamine (**6**) 21
Figure S22. ^{1}H-NMR spectrum of nocardamine (**6**) in DMSO-d6 at 600 MHz. 21
Figure S23. ^{13}C-NMR spectrum of nocardamine (**6**) in DMSO-d6 at 600 MHz. 22
Figure S24. Multiplicity edited HSQC NMR spectrum of nocardamine (**6**) in DMSO-d6 at 600 MHz. 22
Figure S25. ^{1}H-^{1}H COSY spectrum of nocardamine (6) in DMSO-d6 at 600 MHz. 23
Figure S26. HMBC NMR spectrum of nocardamine (**6**) in DMSO-d6 at 600 MHz. 24
Figure S27. HR-ESI-MS spectrum of hexahydro-3-[(4-hydroxyphenyl)methyl]-Pyrrolo[1,2-a]pyrazine-1,4-dione (**7**) 24
Figure S28. ^{1}H-NMR spectrum of hexahydro-3-[(4-hydroxyphenyl)methyl]-Pyrrolo[1,2-a]pyrazine-1,4-dione (**7**) in MeOD at 600 MHz 25
Figure S29. Multiplicity edited HSQC NMR spectrum of hexahydro-3-[(4-hydroxyphenyl)methyl]-Pyrrolo[1,2-a]pyrazine-1,4-dione (**7**) in MeOD at 600 MHz 25
Figure S30. ^{1}H-^{1}H COSY spectrum of hexahydro-3-[(4-hydroxyphenyl)methyl]-Pyrrolo[1,2-a]pyrazine-1,4-dione (**7**) in MeOD at 600 MHz 26

Figure S31. HMBC NMR spectrum of hexahydro-3-[(4-hydroxyphenyl)methyl]-Pyrrolo[1,2-a]pyrazine-1,4-dione (**7**) in MeOD at 600 MHz 26
Figure S32. Molecular network of the molecular families F1, F2, F6-F8, F11 and the single nodes 477.2555 $[M+H]^+$, 555.3857 $[M+H]^+$ and 571.3807 $[M+H]^+$. Nodes of isolated compounds (**1**-**6**) are shown with solid arrowed lines while dereplicated nodes (**8**-**16**) are indicated by dotted arrowed lines. Nodes were represented as pie charts indicating their intensities or percentages in the methanol (red nodes), ethyl acetate (blue nodes) and dichloromethane (green nodes) extracts of the fermentation broth of *K. speibonae* strain SK5. Nodes with asterisk (*) indicate dehydroxylated desferrioxamines. 27
Figure S33. A MN showing a link between nodes *di*desoxy-desferrioxamine D_1 **8** (571.3807 $[M+H]^+$) and desoxy-desferrioxamine D_1 **2** (587.3754 $[M+H]^+$) when default parameters was used in generating the MN on the GNPS platform. 28
Figure S34. MS/MS spectrum of desoxy-desferrioxamine D_1 **2** (587.3754 $[M + H]^+$) with annotation in the structure........ 29
Figure S35. MS/MS spectrum of *di*desoxy-desferrioxamine D_1 **8** (571.3807 $[M + H]^+$) with annotation in the structure. 30
Figure S36. MS/MS spectrum of speibonoxamine **1** (555.3857 $[M + H]^+$) with annotation in the structure. 31
Figure S37. MS/MS spectrum of desferrioxamine B **4** (561.3606 $[M + H]^+$) with annotation in the structure........ 32
Figure S38. MS/MS spectrum desoxy-desferrioxamine B **9** (545.3654 $[M + H]^+$) with annotation in the structure........ 33
Figure S39. MS/MS spectrum *di*desoxy-desferrioxamine B **10** (529.3702 $[M + H]^+$) with annotation in the structure........ 34

List of Table

Table S1. Compounds isolated and tentatively identified in the molecular cluster and the MS chromatogram of the fermentation broth of *K. speibonae* strain SK5 with their corresponding masses, molecular formulae (MF), double bond equivalent (DBE), retention time (Rt) and mass error (ID (Δ ppm)) 35

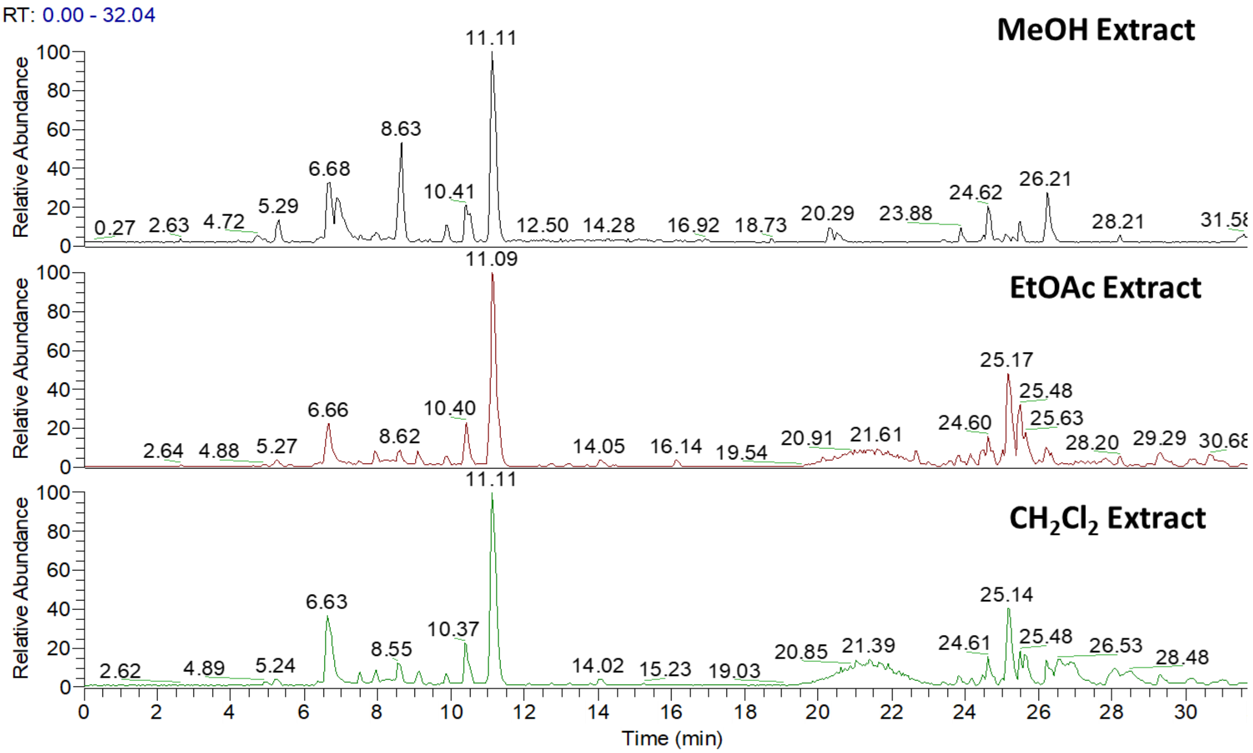

Figure S1. MS window of HPLC-DAD/HRESIMS profiles of MeOH (top), EtOAC (middle) and CH_2Cl_2 (bottom) extracts of the fermentation broth of *Kribbella speibonae* strain SK5 cultured in ISP2 liquid medium.

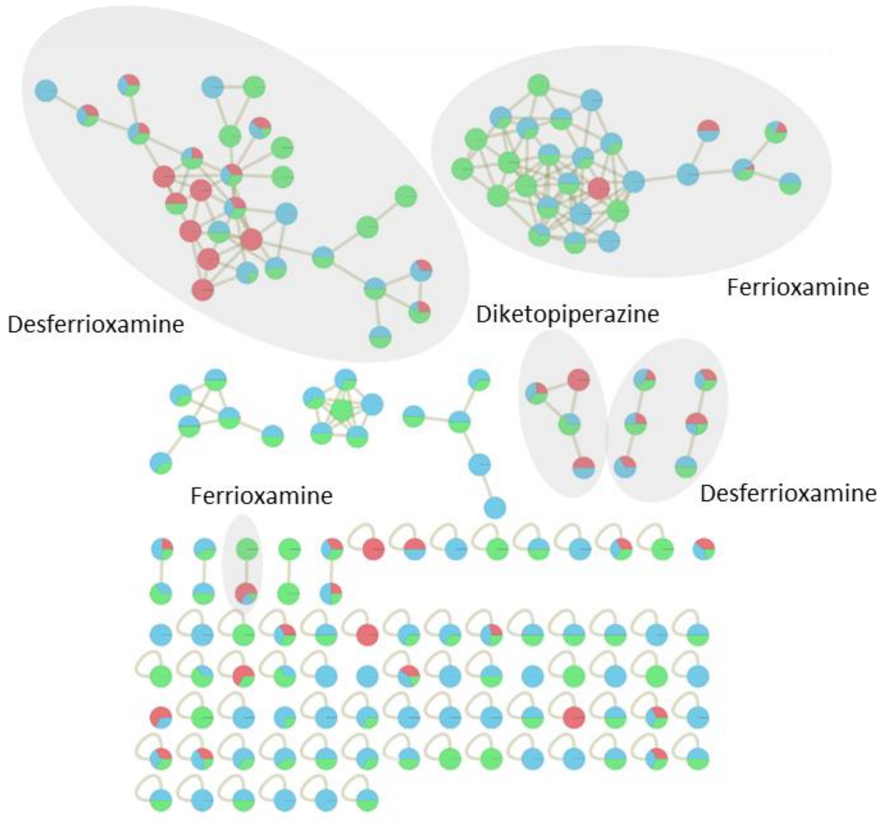

Figure S2. GNPS molecular network of the methanol (red nodes), ethyl acetate (blue nodes) and dichloromethane (green nodes) extracts of the fermentation broth of *K. speibonae* strain SK5. The molecular network contains 13 families with the highlighted families (desferrioxamines, ferrioxamines and diketopiperazines) containing some annotated nodes.

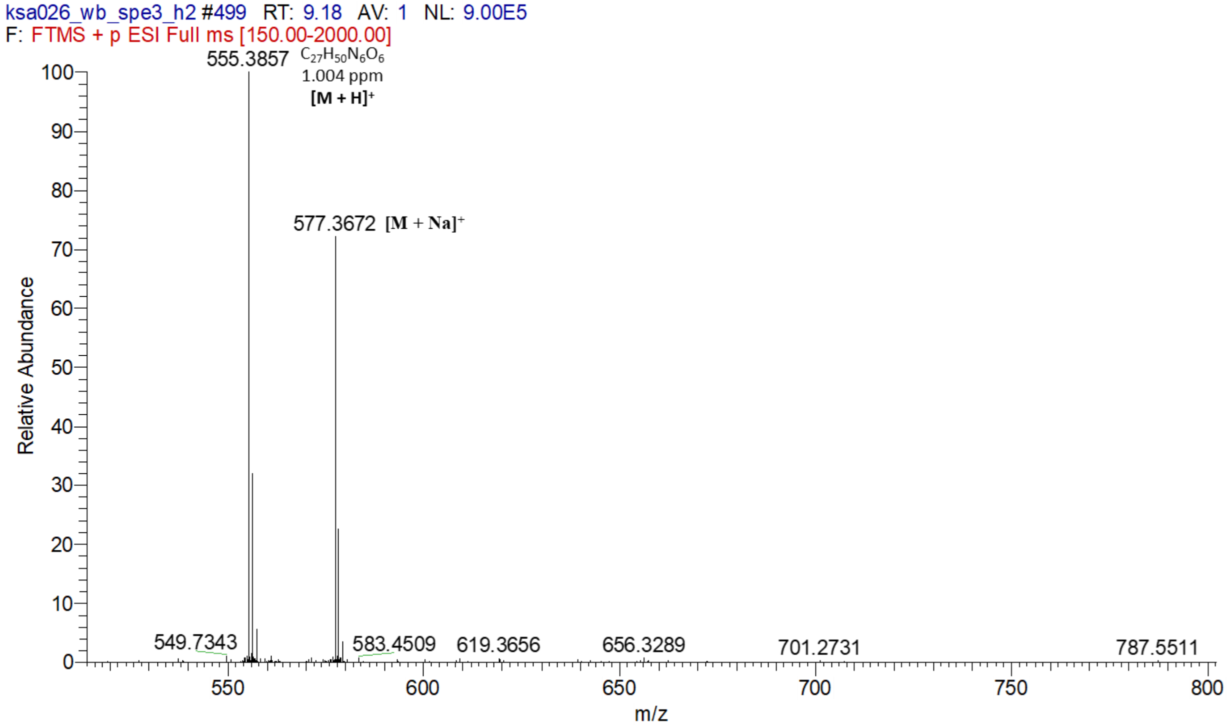

Figure S3. HR-ESI-MS spectrum of speibonoxamine (**1**)

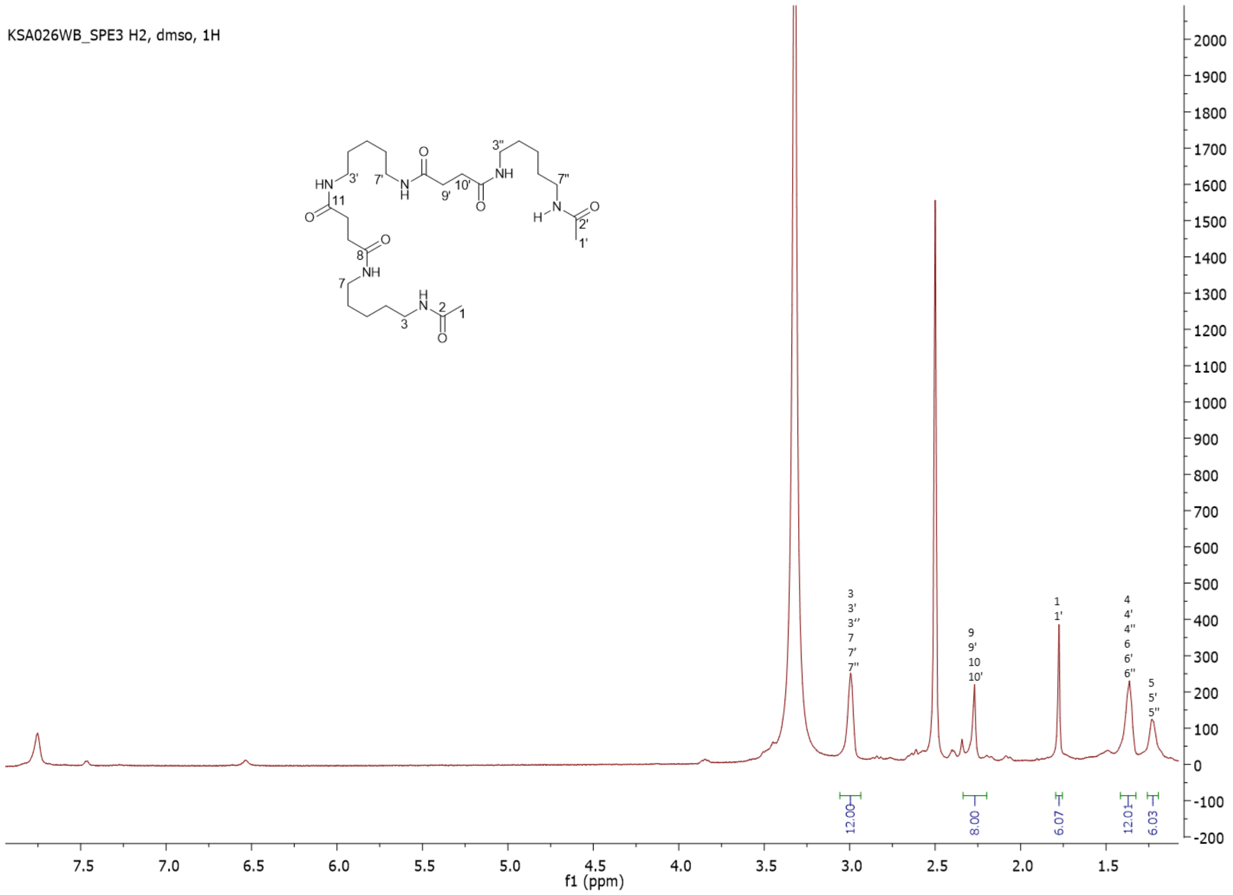

Figure S4. ^{1}H-NMR spectrum of speibonoxamine (**1**) in DMSO-d6 at 600 MHz.

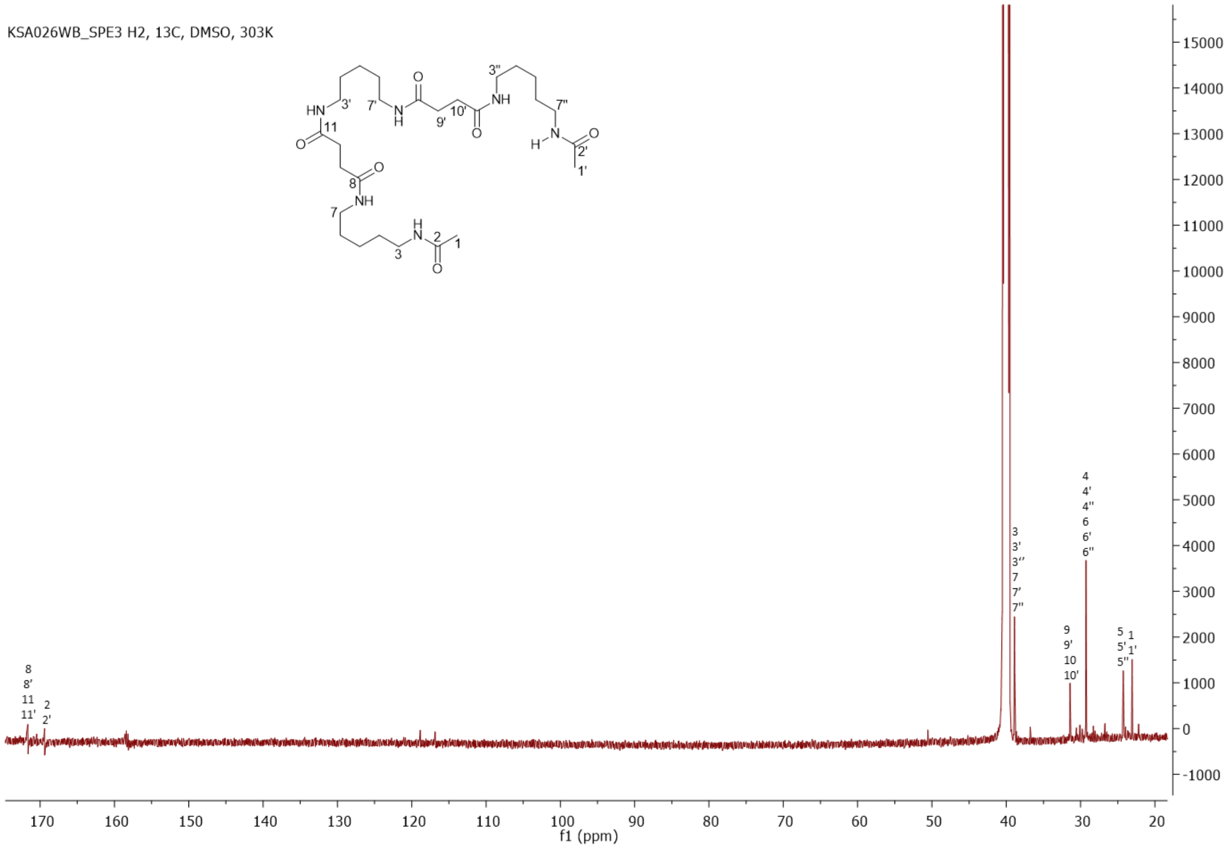

Figure S5. ^{13}C-NMR spectrum of speibonoxamine (**1**) in DMSO-d6 at 600 MHz.

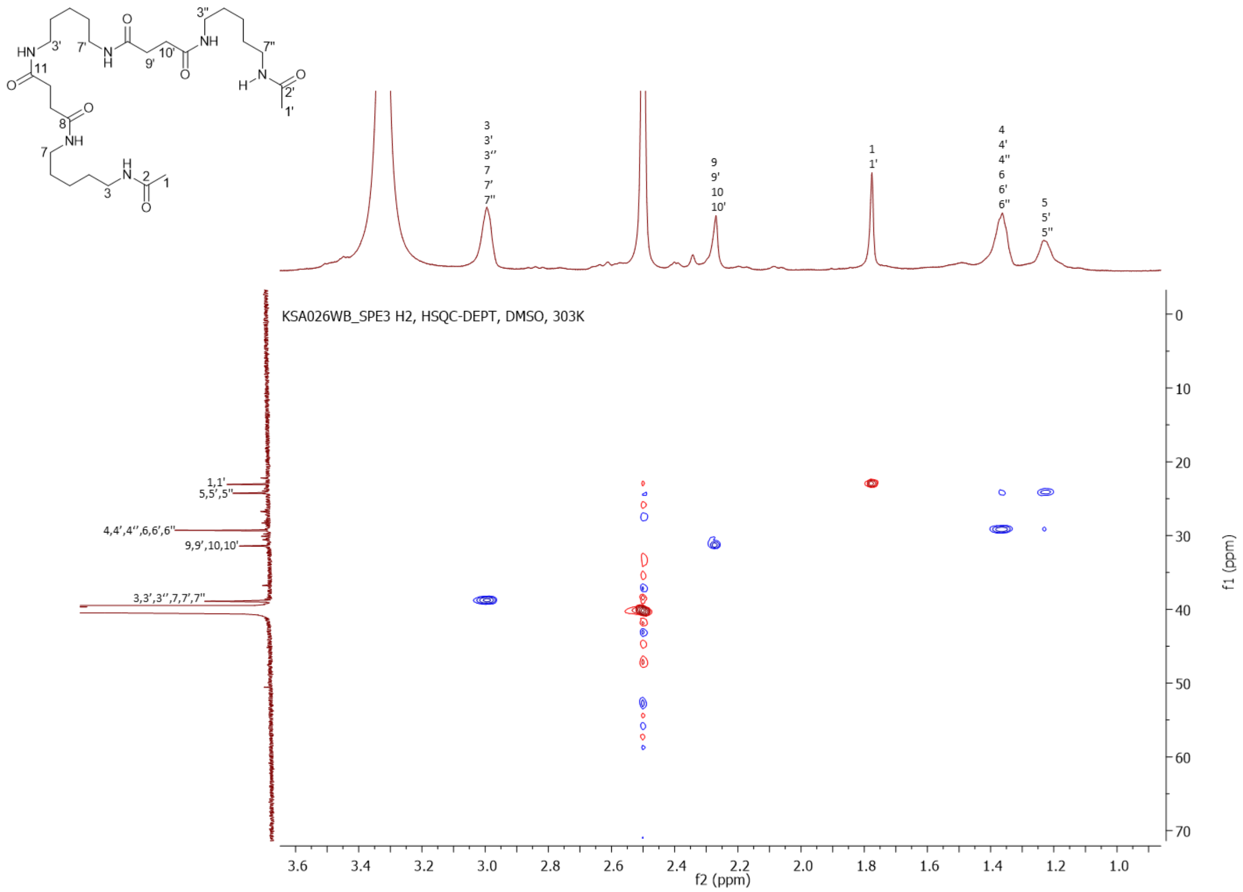

Figure S6. Multiplicity edited HSQC NMR spectrum of speibonoxamine (**1**) in DMSO-d6 at 600 MHz.

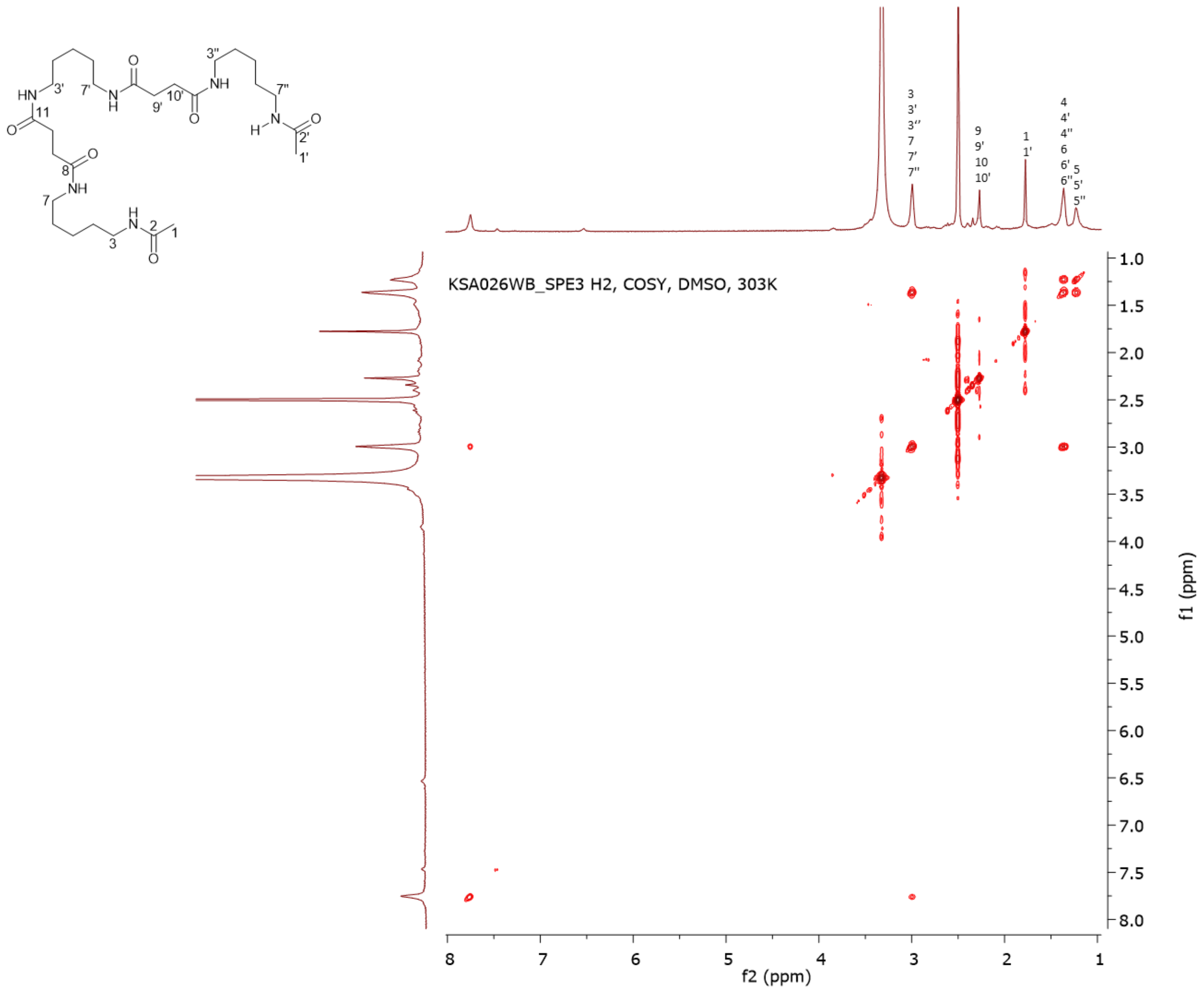

Figure S7. ^{1}H-^{1}H COSY spectrum of speibonoxamine (**1**) in DMSO-d6 at 600 MHz.

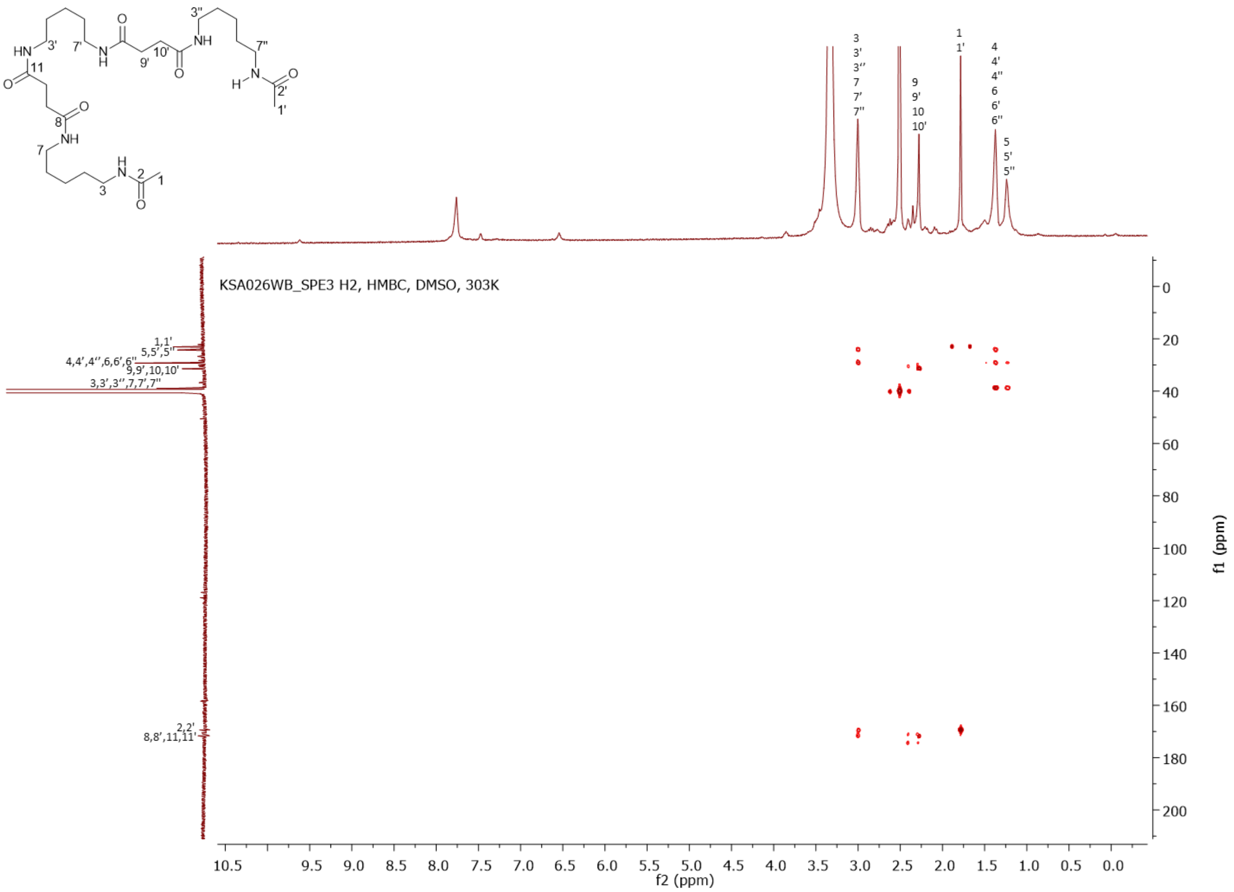

Figure S8. HMBC NMR spectrum of speibonoxamine (**1**) in DMSO-d6 at 600 MHz.

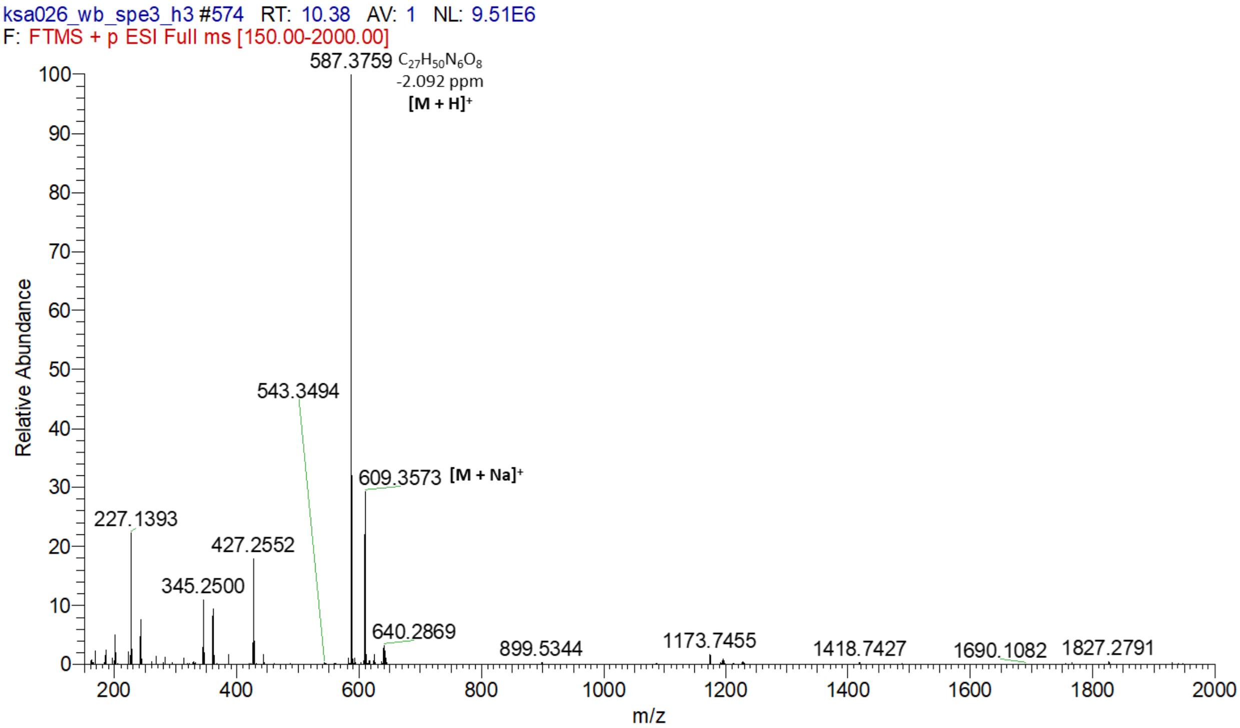

Figure S9. HR-ESI-MS spectrum of desoxy-desferrioxmaine D_1 (**2**)

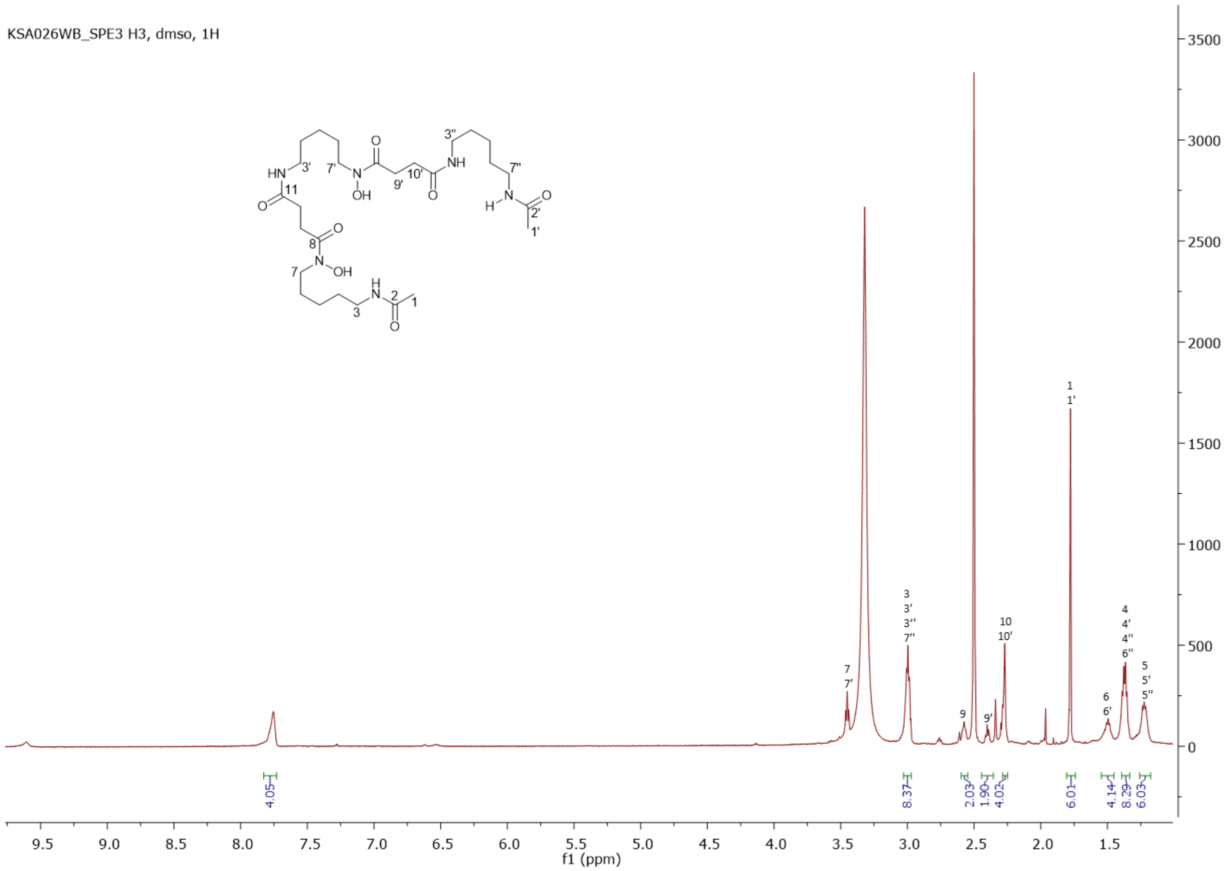

Figure S10. ^{1}H-NMR spectrum of desoxy-desferrioxmaine D_1 (**2**) in DMSO-d6 at 600 MHz.

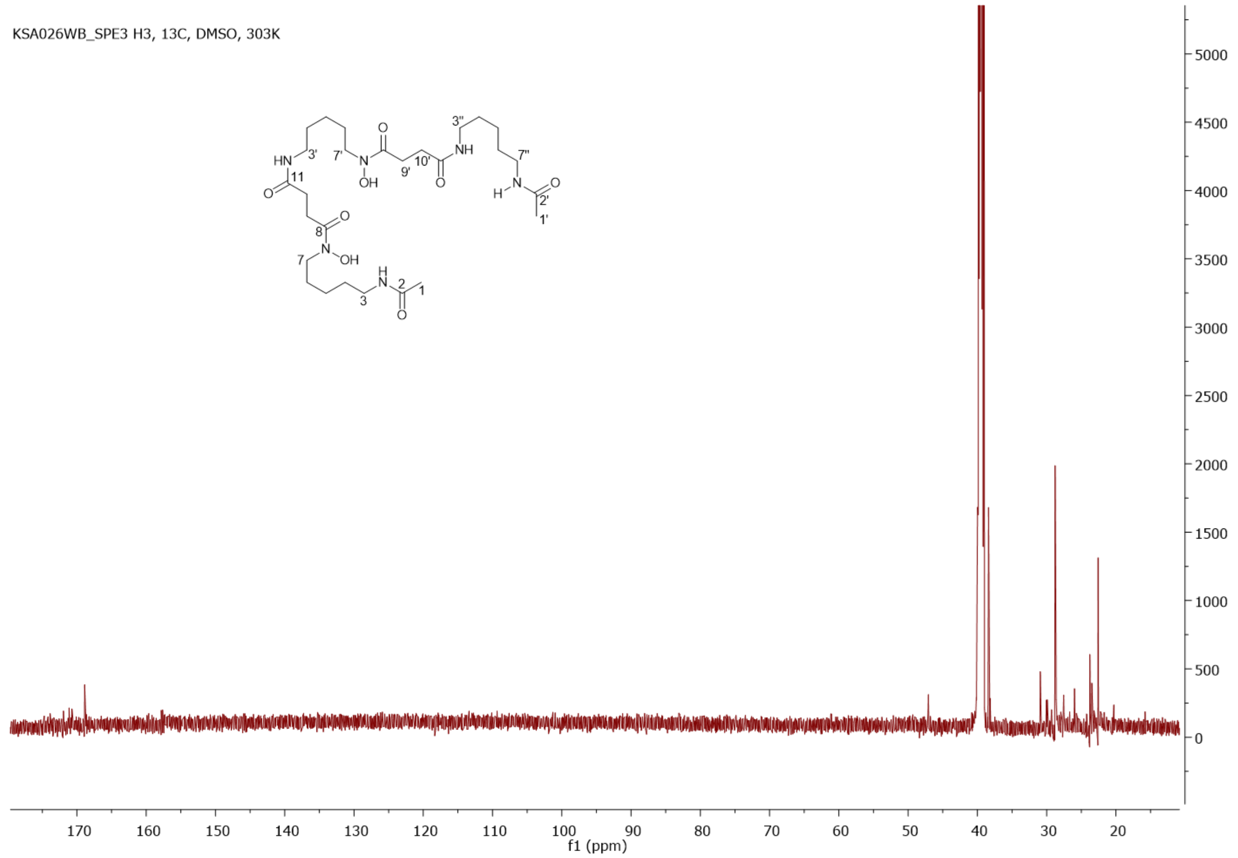

Figure S11. ^{13}C-NMR spectrum of desoxy-desferrioxmaine D_1 (**2**) in DMSO-d6 at 600 MHz.

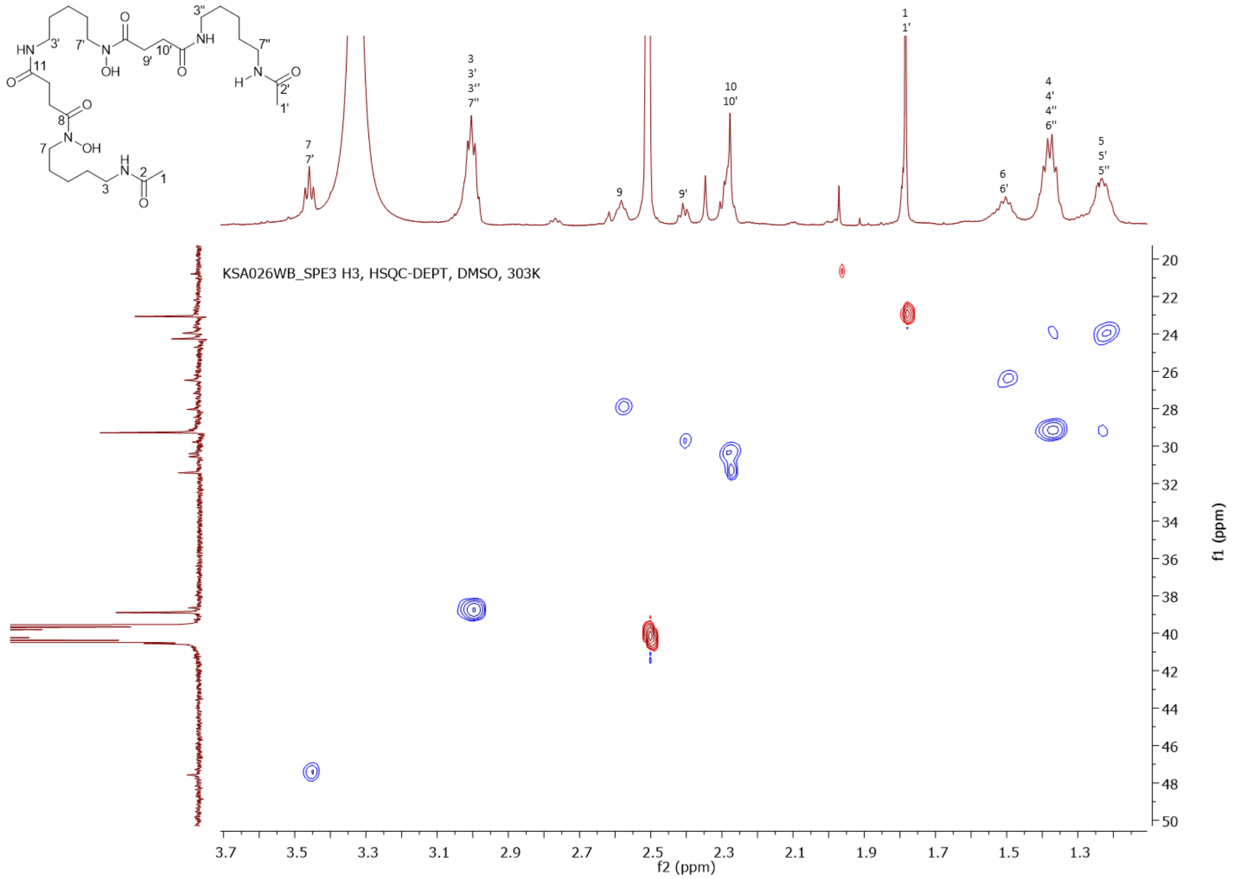

Figure S12. Multiplicity edited HSQC NMR spectrum of desoxy-desferrioxmaine D_1 (**2**) in DMSO-d6 at 600 MHz.

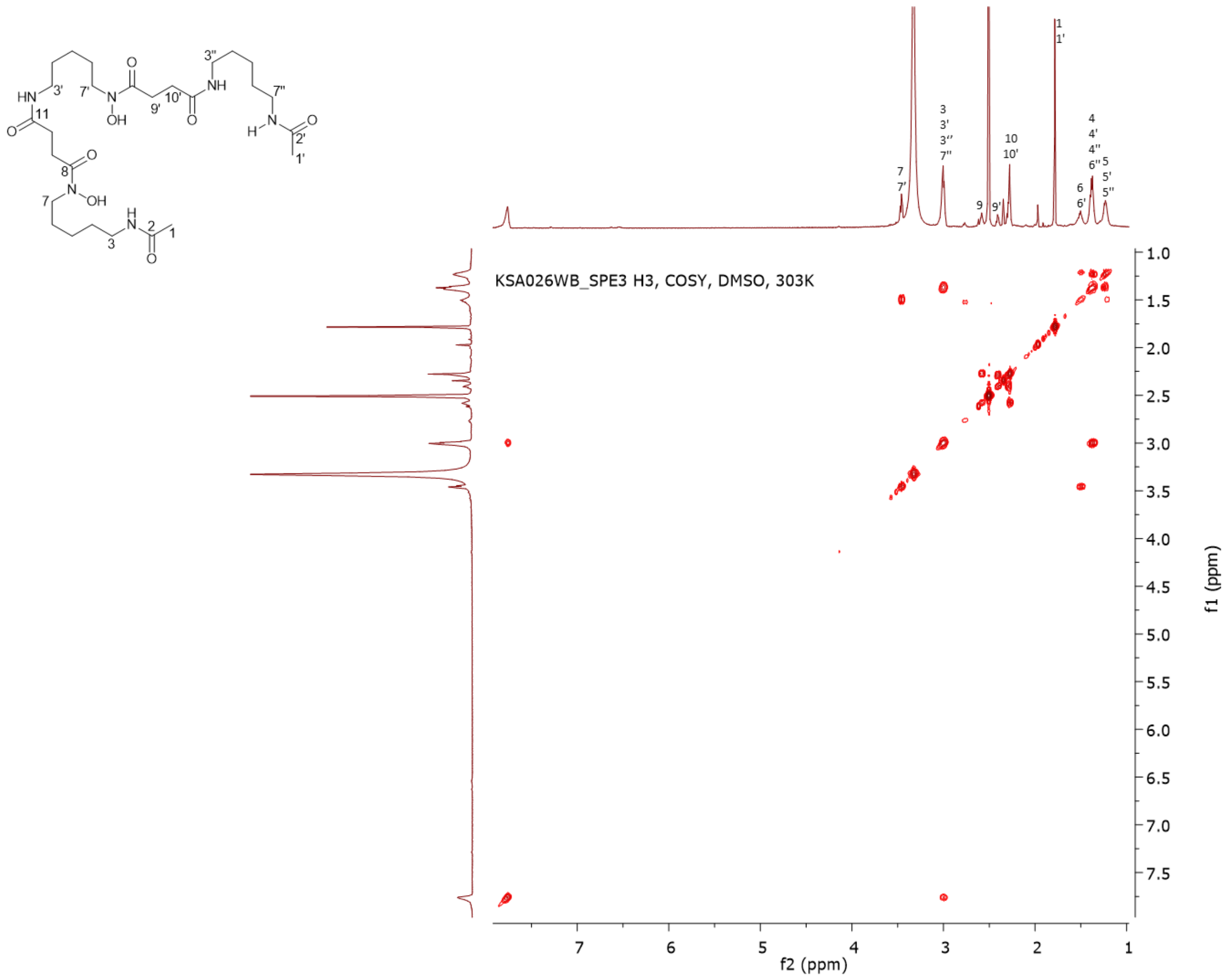

Figure S13. ^{1}H-^{1}H COSY spectrum of desoxy-desferrioxmaine D_1 (**2**) in DMSO-d6 at 600 MHz.

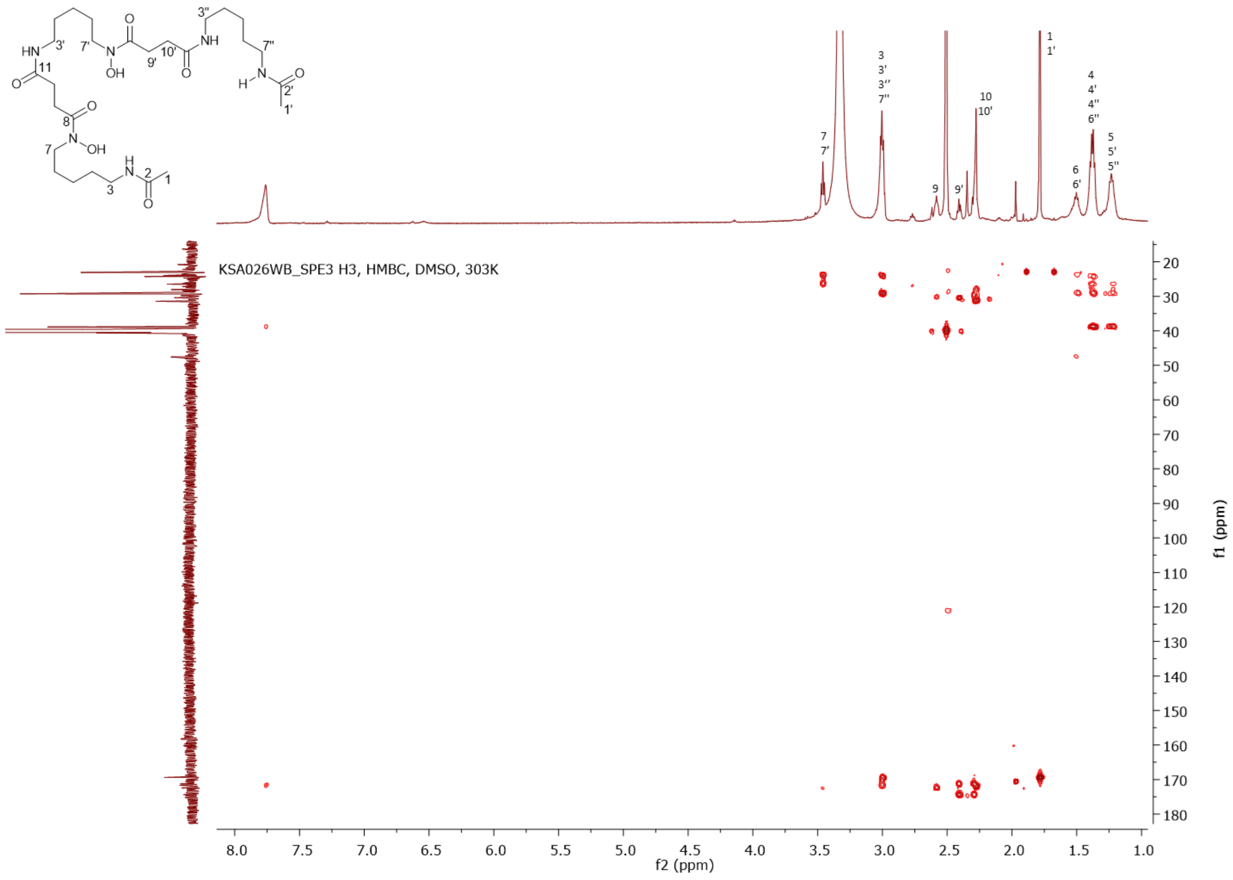

Figure S14. HMBC NMR spectrum of desoxy-desferrioxmaine D_1 (**2**) in DMSO-d6 at 600 MHz.

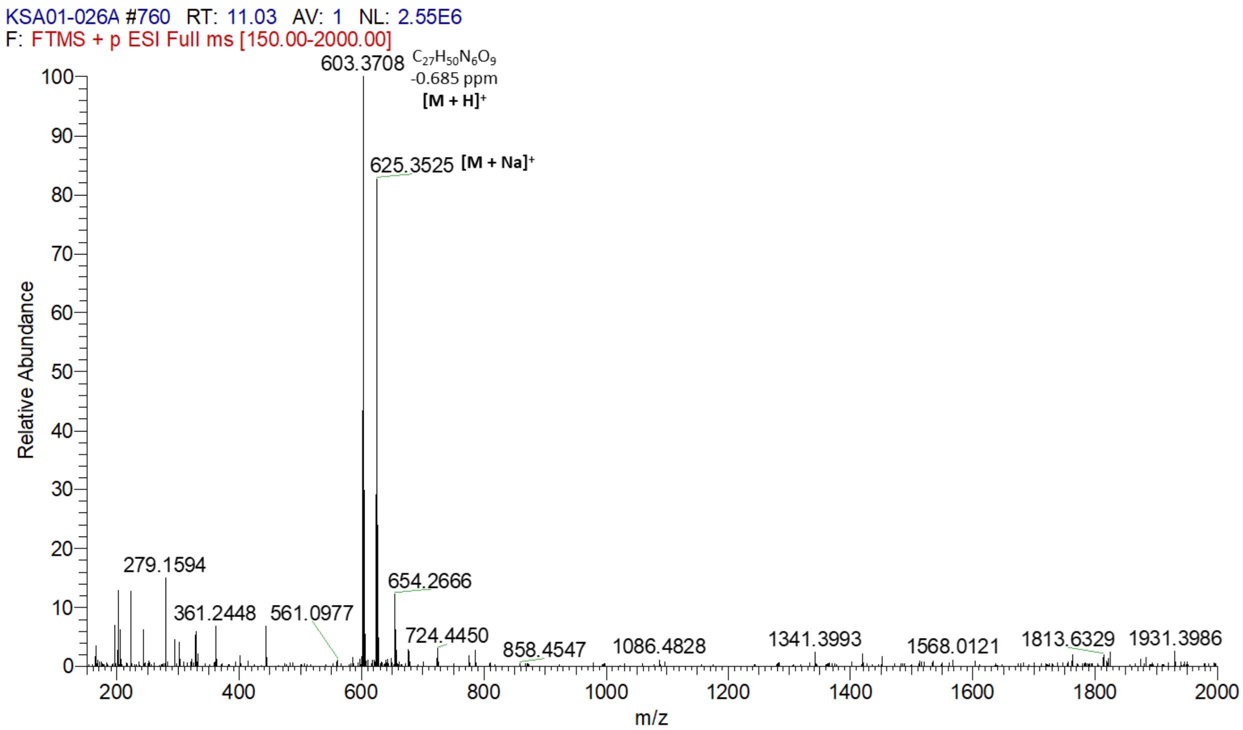

Figure S15. HR-ESI-MS spectrum of desferrioxamine D_1 (**3**)

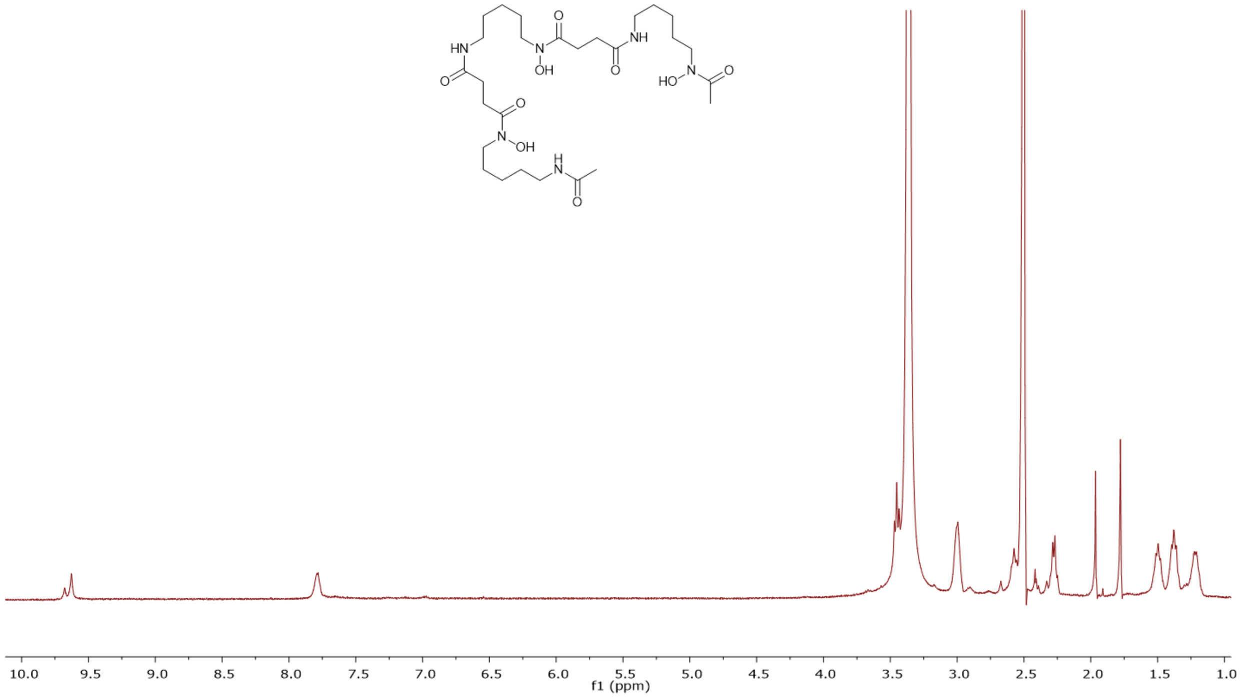

Figure S16. ^{1}H-NMR spectrum of desferrioxamine D_1 (**3**) in DMSO-d6 at 600 MHz.

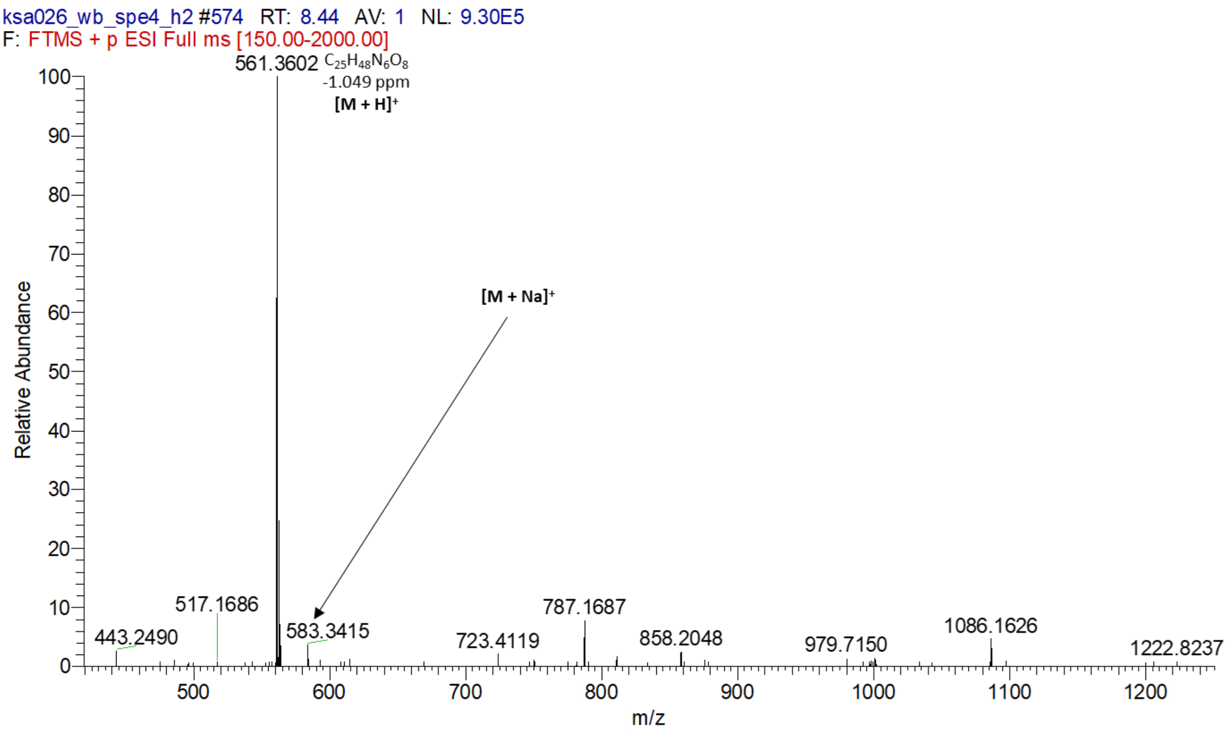

Figure S17. HR-ESI-MS spectrum of desferrioxamine B (**4**)

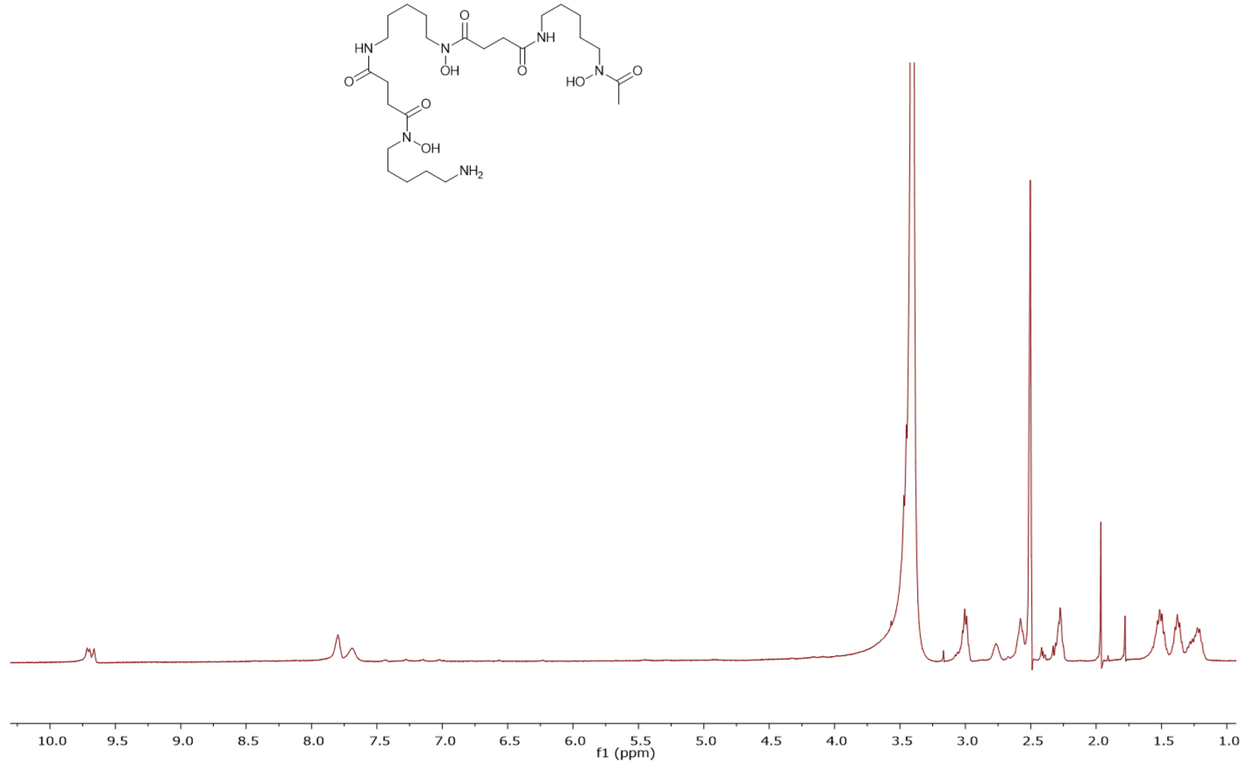

Figure S18. ^{1}H-NMR spectrum of desferrioxamine B (**4**) in DMSO-d6 at 600 MHz.

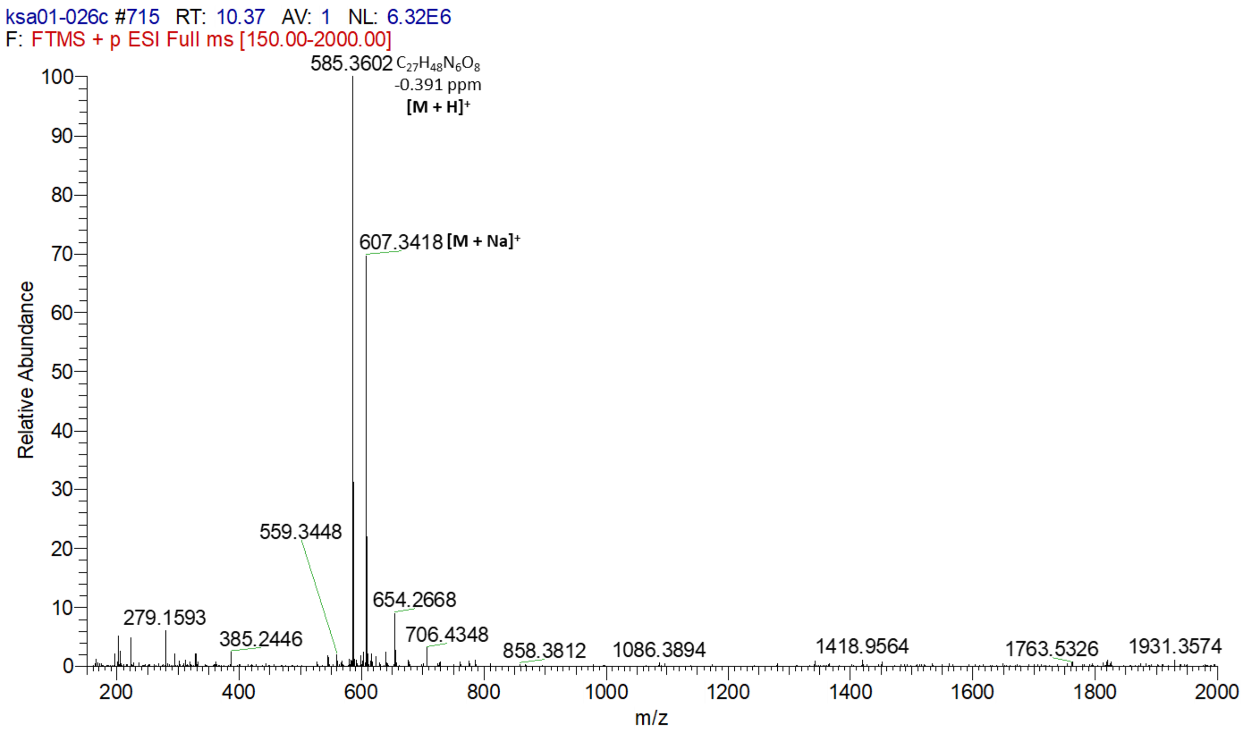

Figure S19. HR-ESI-MS spectrum of desoxynocardamine (**5**)

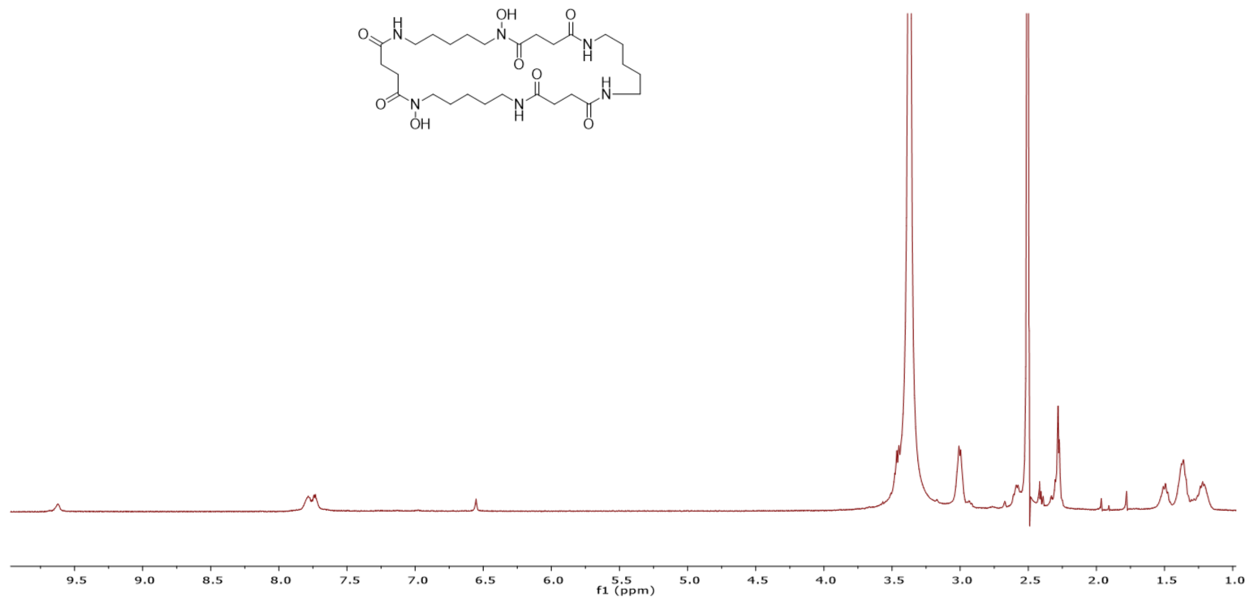

Figure S20. ^{1}H-NMR spectrum of desoxynocardamine (**5**) in DMSO-d6 at 600 MHz.

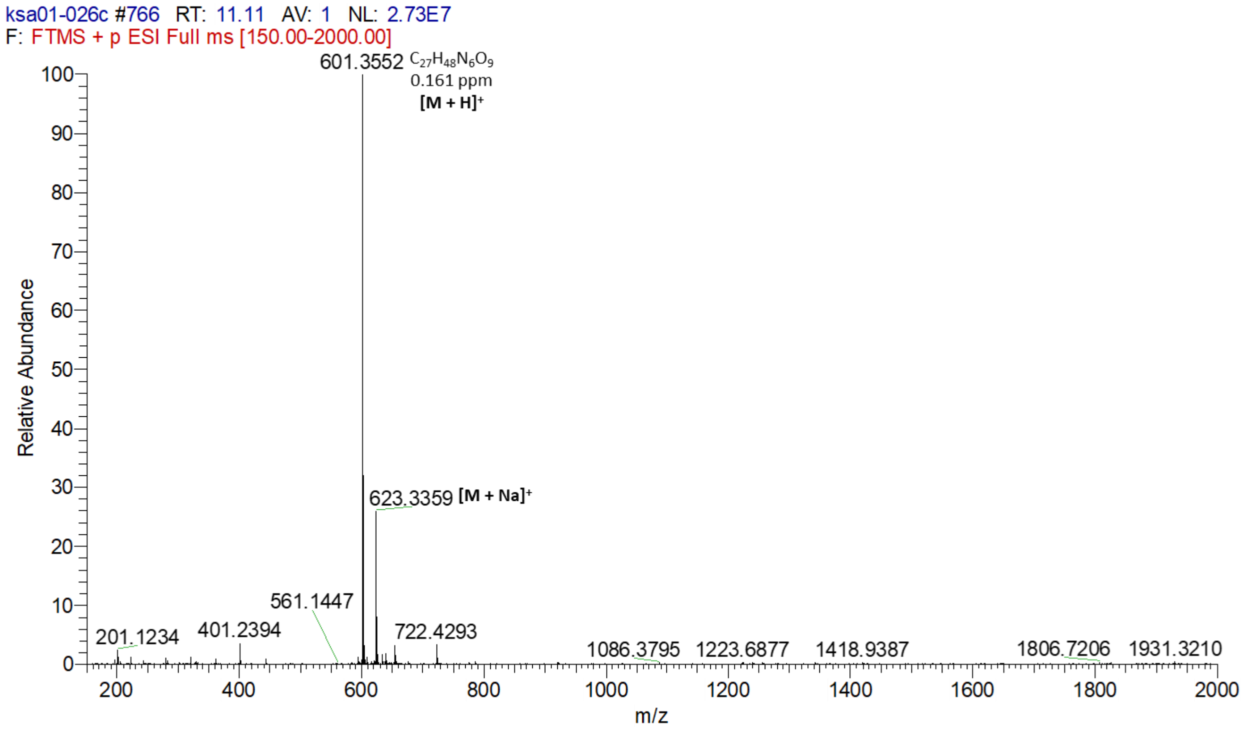

Figure S21. HR-ESI-MS spectrum of nocardamine (**6**)

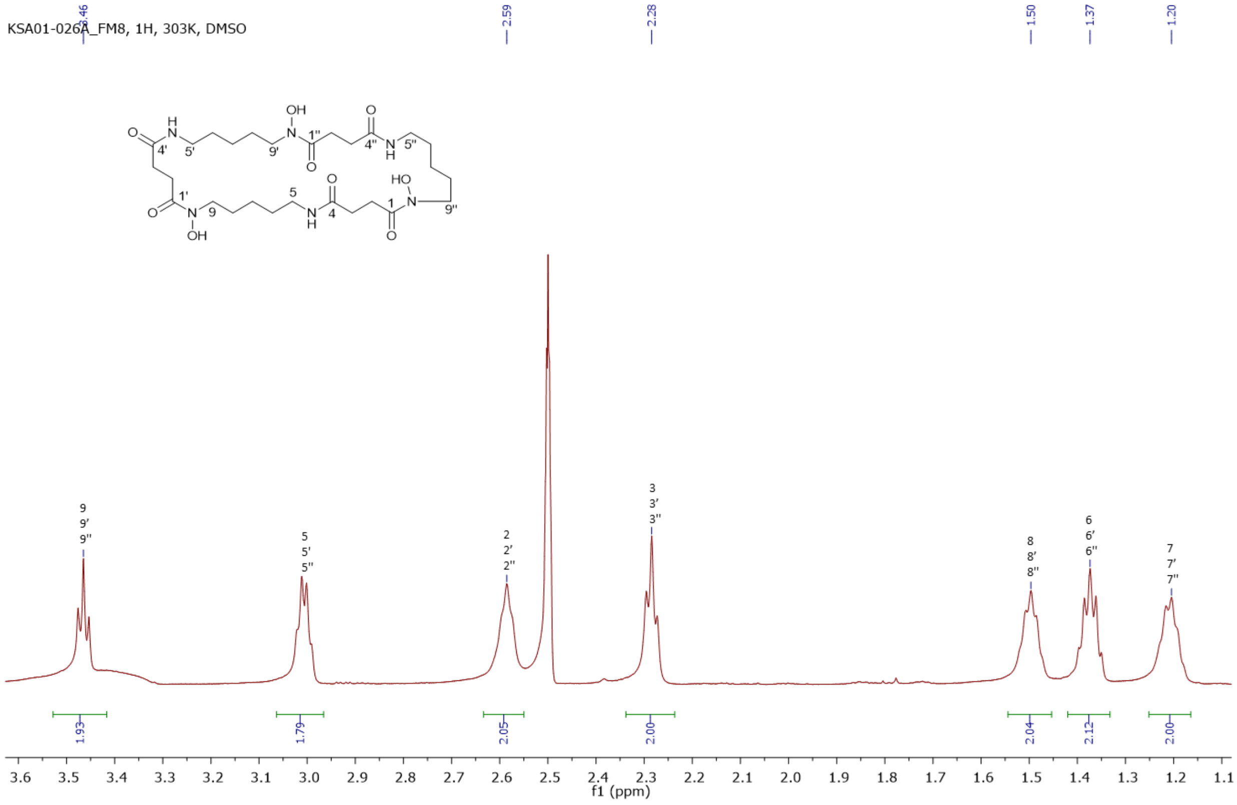

Figure S22. ^{1}H-NMR spectrum of nocardamine (**6**) in DMSO-d6 at 600 MHz.

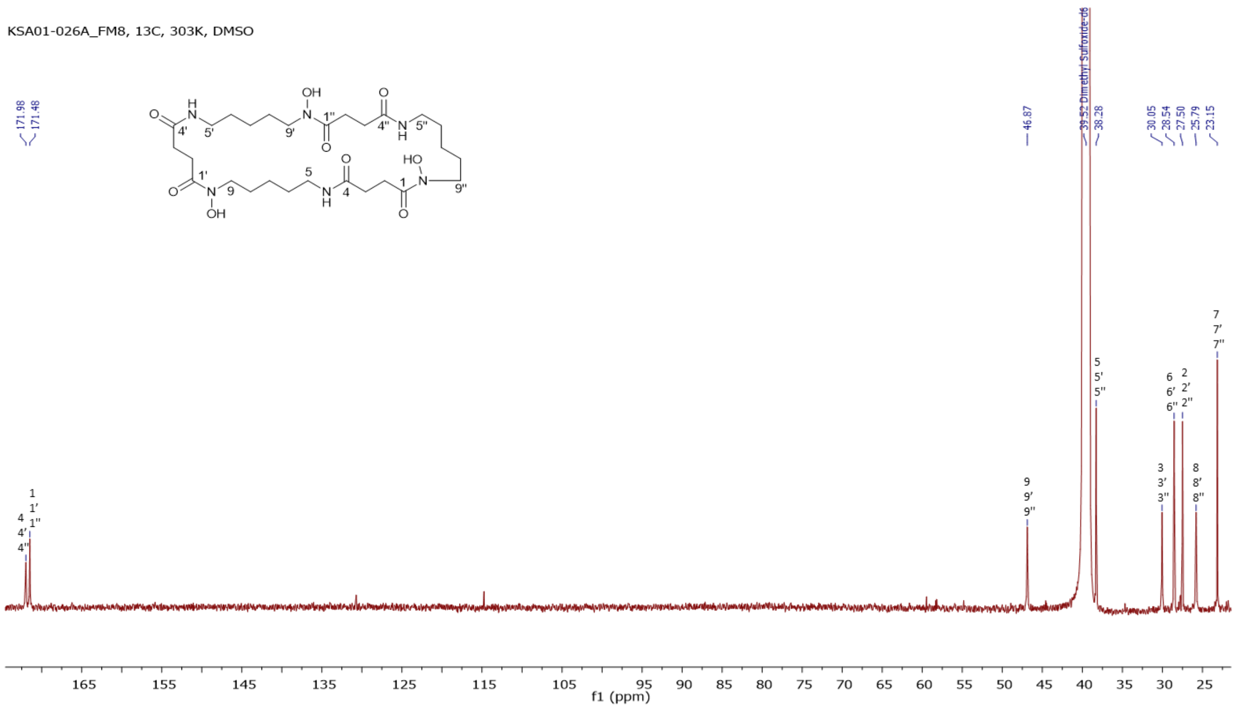

Figure S23. ^{13}C-NMR spectrum of nocardamine (**6**) in DMSO-d6 at 600 MHz.

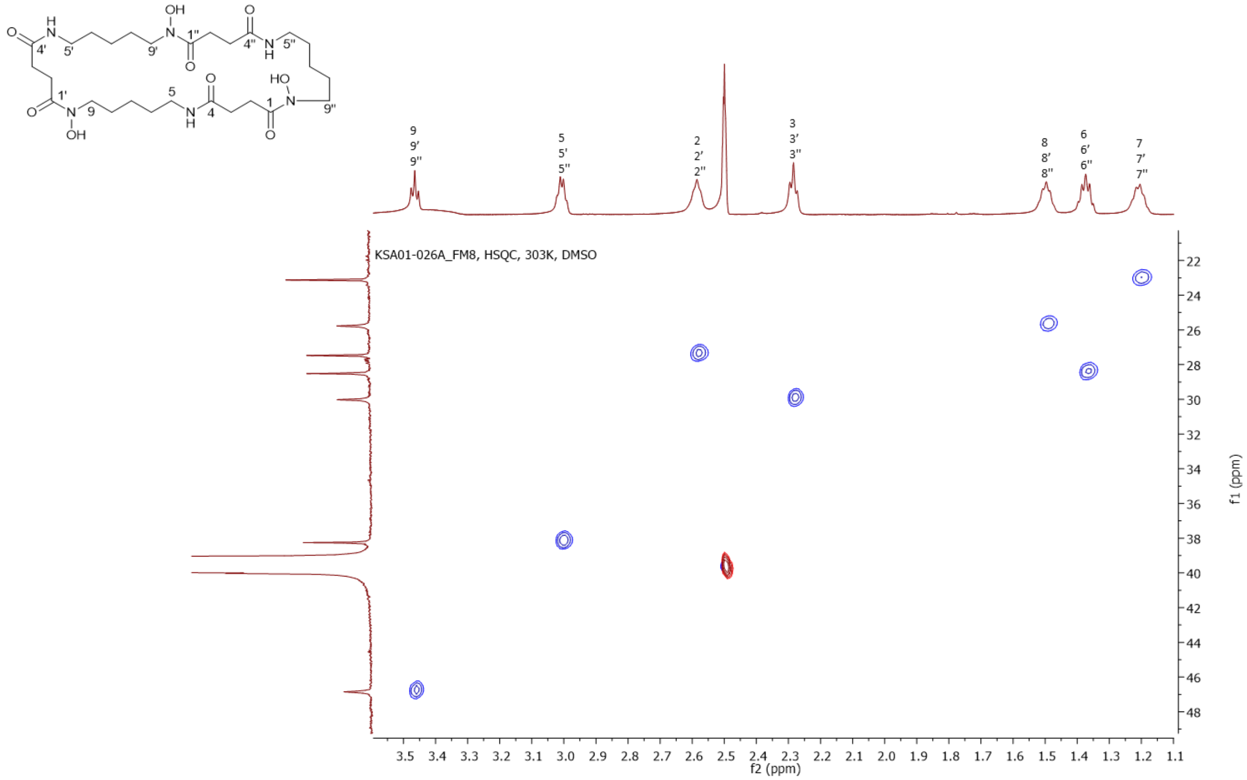

Figure S24. Multiplicity edited HSQC NMR spectrum of nocardamine (**6**) in DMSO-d6 at 600 MHz.

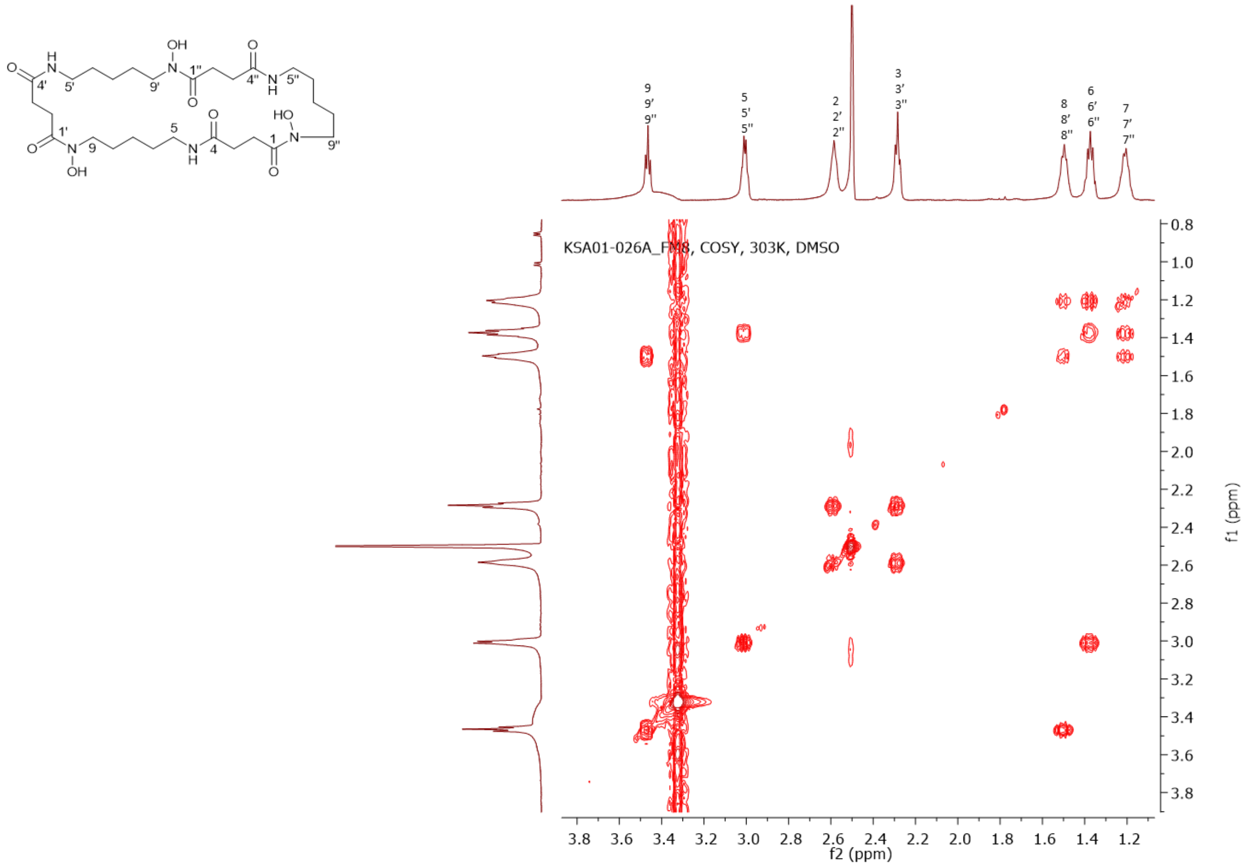

Figure S25. ^{1}H-^{1}H COSY spectrum of nocardamine (6) in DMSO-d6 at 600 MHz.

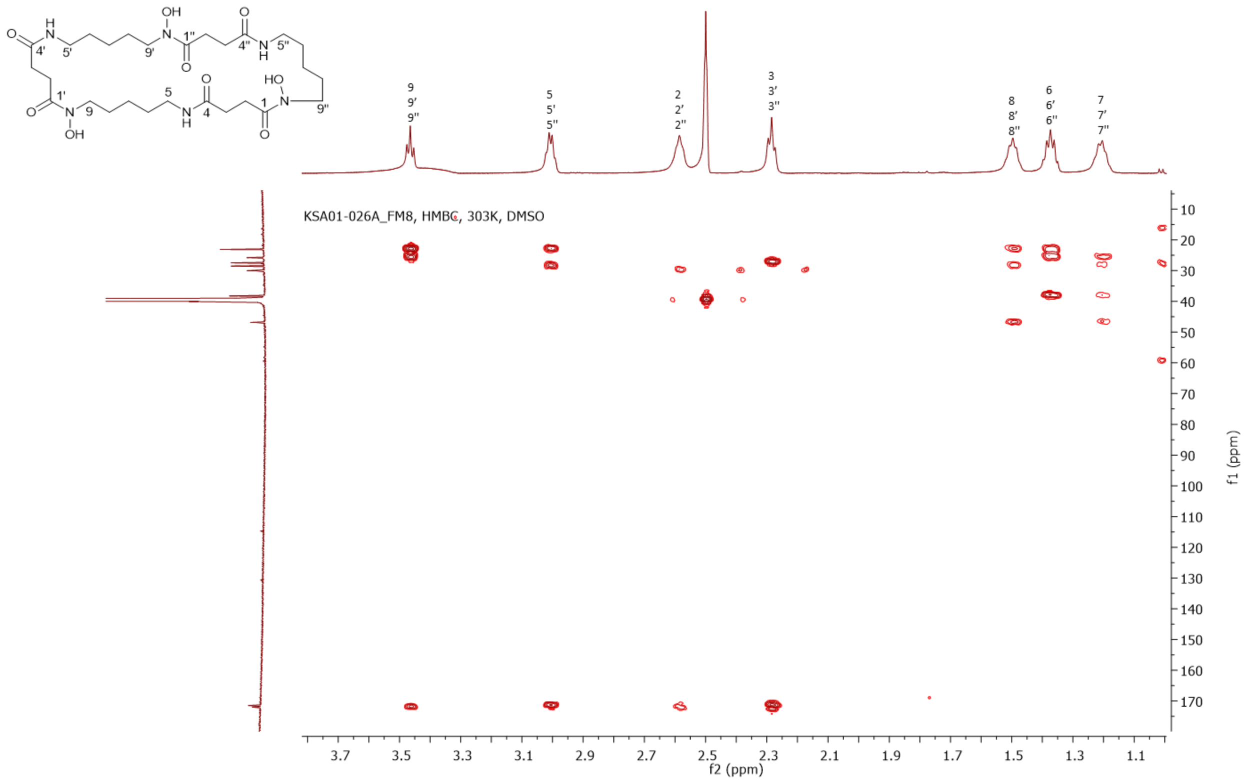

Figure S26. HMBC NMR spectrum of nocardamine (**6**) in DMSO-d6 at 600 MHz.

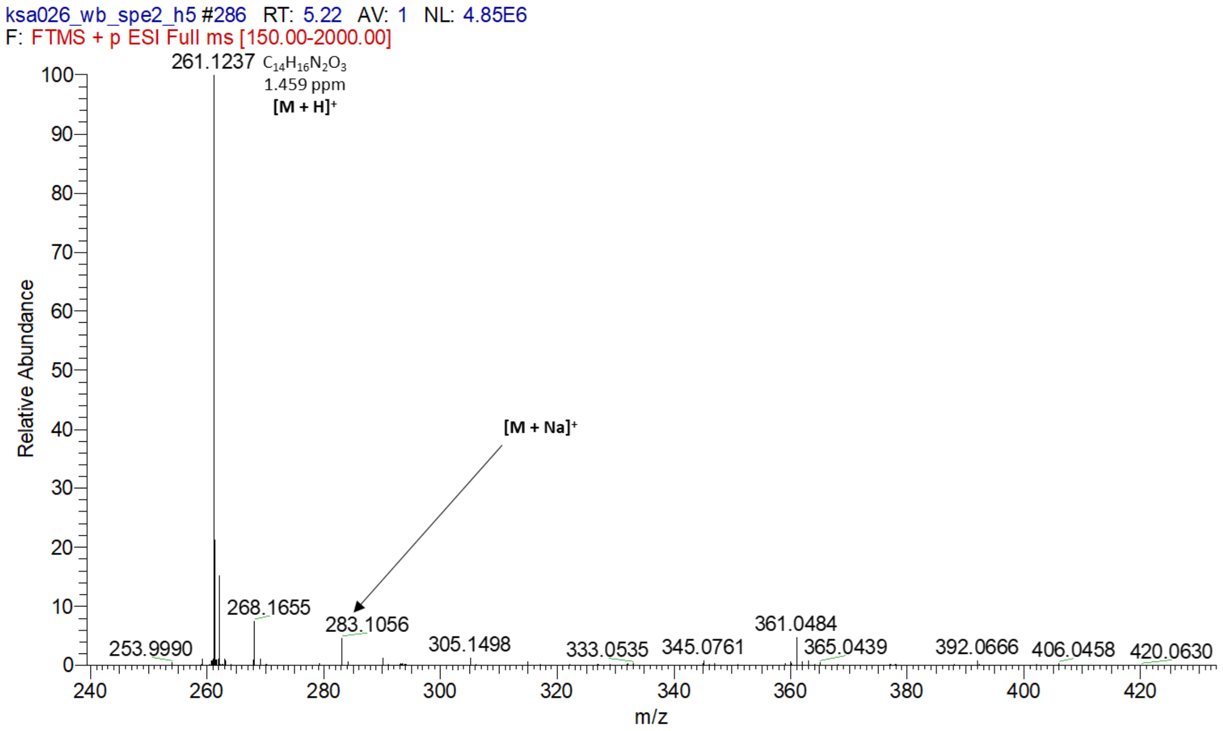

Figure S27. HR-ESI-MS spectrum of hexahydro-3-[(4-hydroxyphenyl)methyl]-Pyrrolo[1,2-a]pyrazine-1,4-dione (**7**)

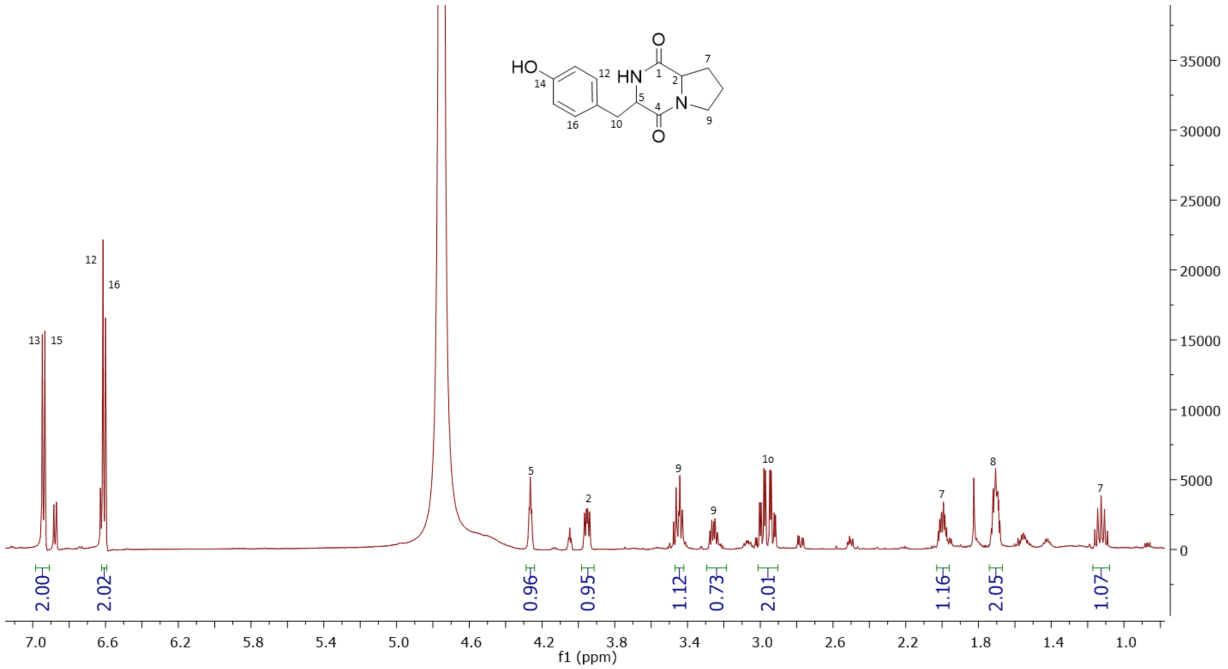

Figure S28. ¹H-NMR spectrum of hexahydro-3-[(4-hydroxyphenyl)methyl]-Pyrrolo[1,2-a]pyrazine-1,4-dione (**7**) in MeOD at 600 MHz

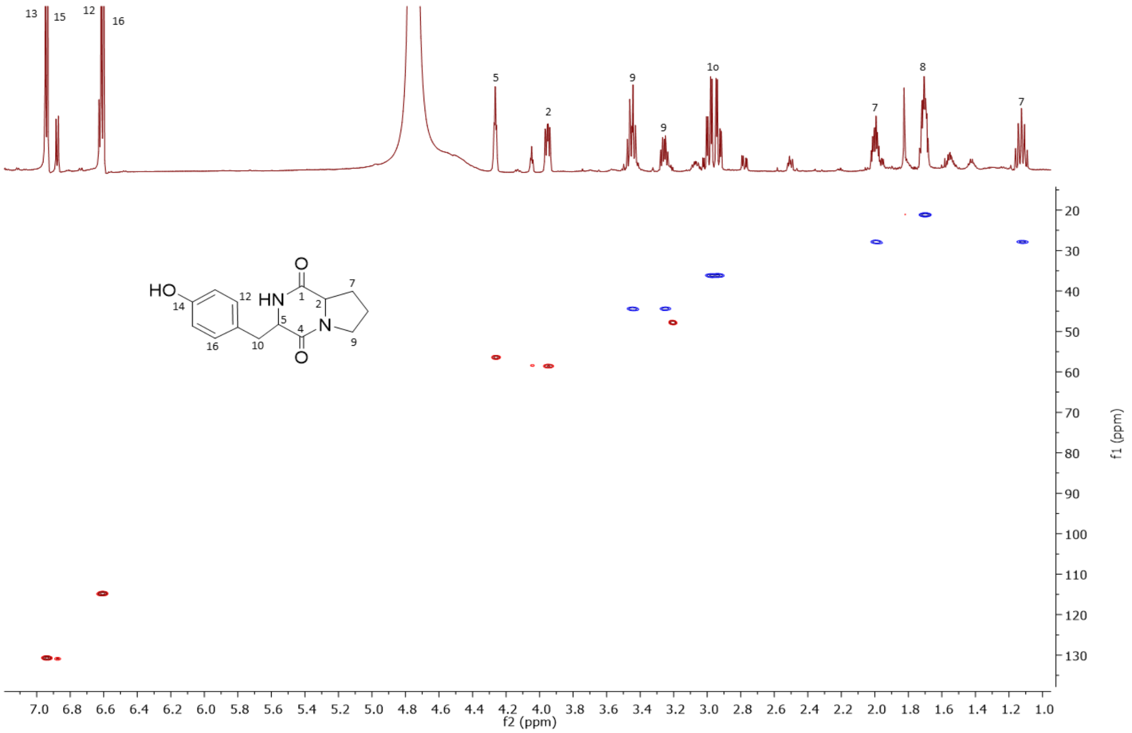

Figure S29. Multiplicity edited HSQC NMR spectrum of hexahydro-3-[(4-hydroxyphenyl)methyl]-Pyrrolo[1,2-a]pyrazine-1,4-dione (**7**) in MeOD at 600 MHz

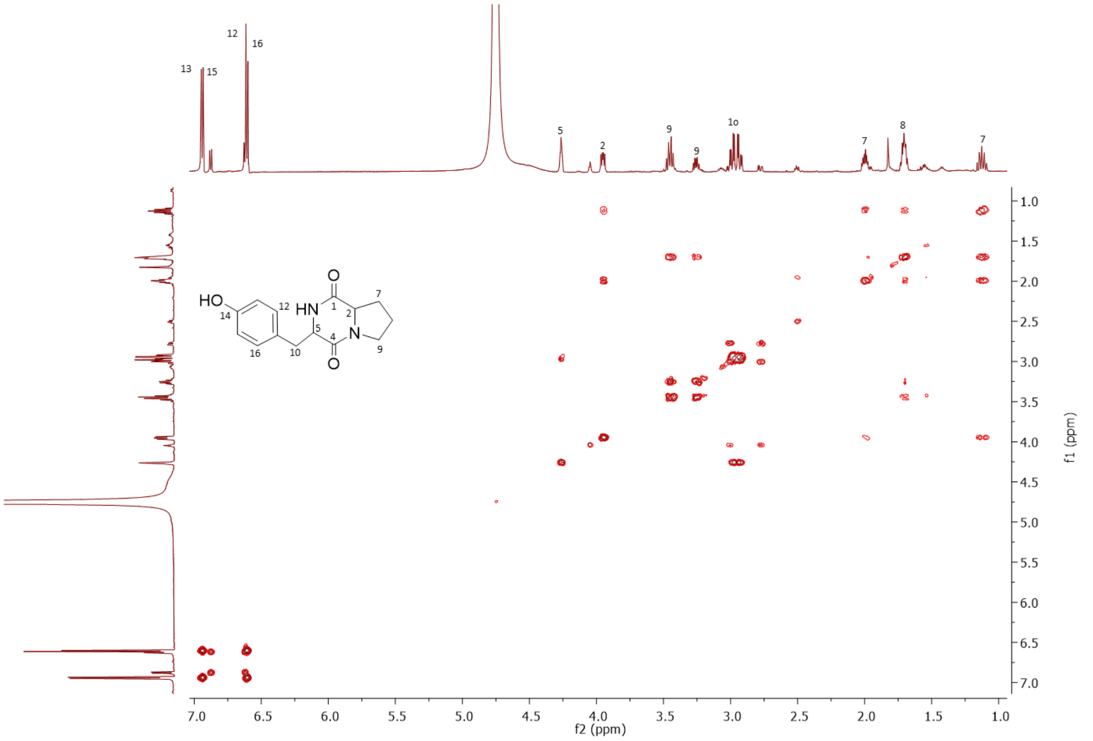

Figure S30. ^{1}H-^{1}H COSY spectrum of hexahydro-3-[(4-hydroxyphenyl)methyl]-Pyrrolo[1,2-a]pyrazine-1,4-dione (**7**) in MeOD at 600 MHz

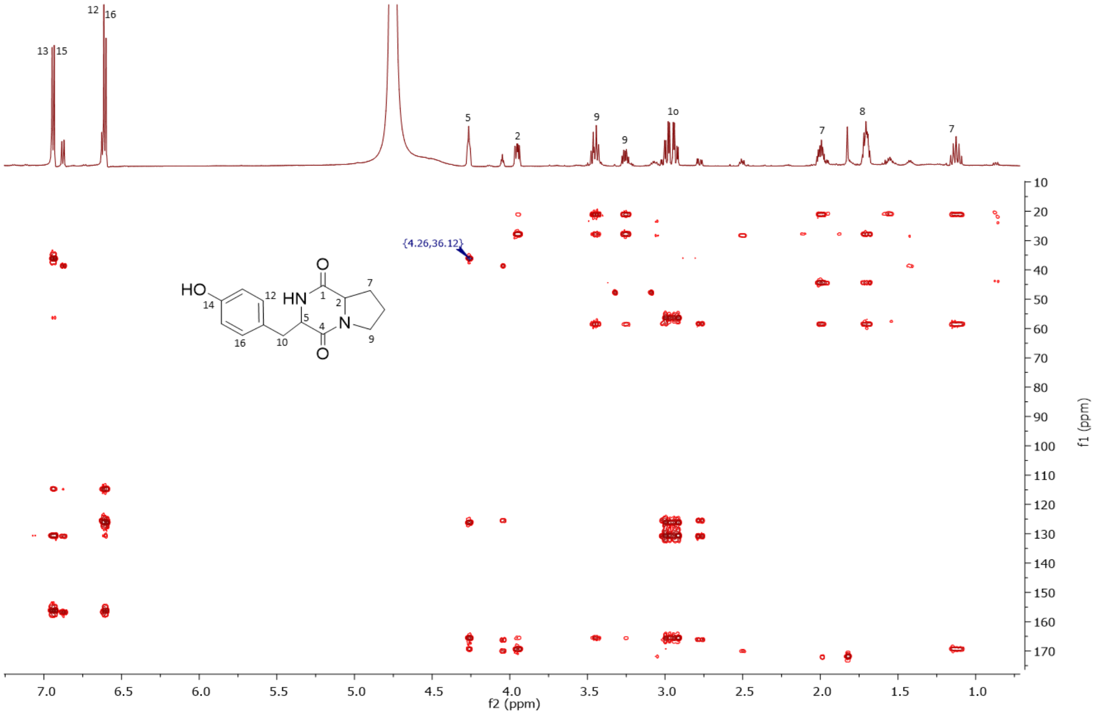

Figure S31. HMBC NMR spectrum of hexahydro-3-[(4-hydroxyphenyl)methyl]-Pyrrolo[1,2-a]pyrazine-1,4-dione (**7**) in MeOD at 600 MHz

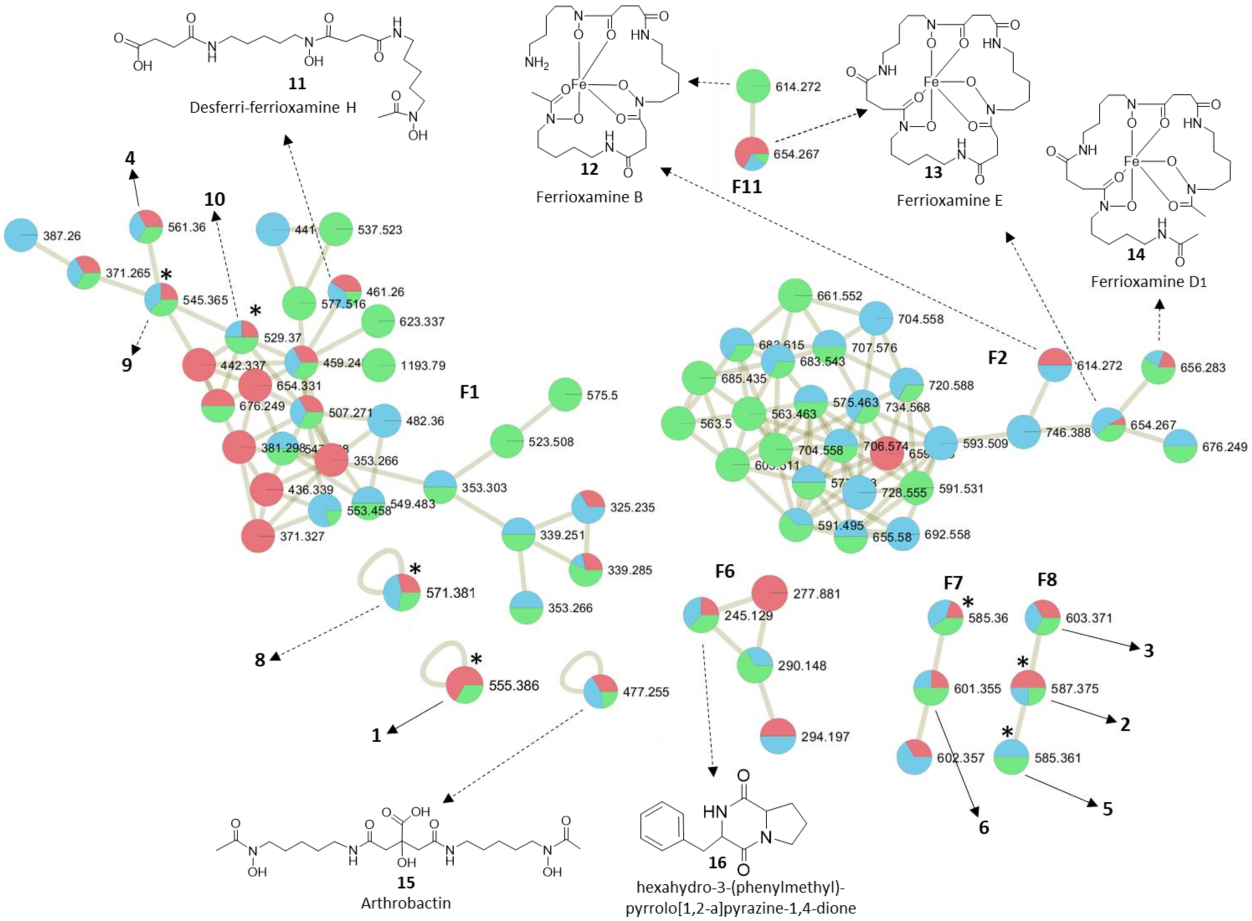

Figure S32. Molecular network of the molecular families F1, F2, F6-F8, F11 and the single nodes 477.2555 $[M+H]^+$, 555.3857 $[M+H]^+$ and 571.3807 $[M+H]^+$. Nodes of isolated compounds (**1**-**6**) are shown with solid arrowed lines while dereplicated nodes (**8**-**16**) are indicated by dotted arrowed lines. Nodes were represented as pie charts indicating their intensities or percentages in the methanol (red nodes), ethyl acetate (blue nodes) and dichloromethane (green nodes) extracts of the fermentation broth of *K. speibonae* strain SK5. Nodes with asterisk (*) indicate dehydroxylated desferrioxamines.

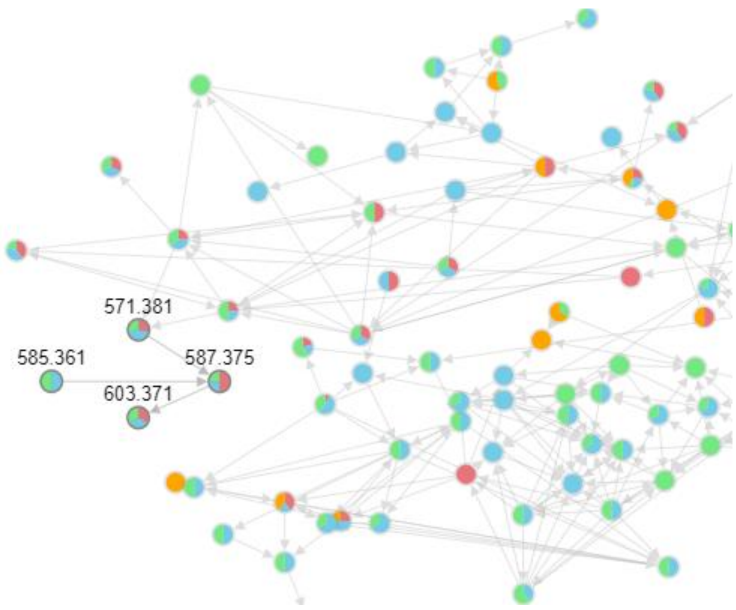

Figure S33. A MN showing a link between nodes *di*desoxy-desferrioxamine D_1 **8** (571.3807 $[M+H]^+$) and desoxy-desferrioxamine D_1 **2** (587.3754 $[M+H]^+$) when default parameters was used in generating the MN on the GNPS platform.

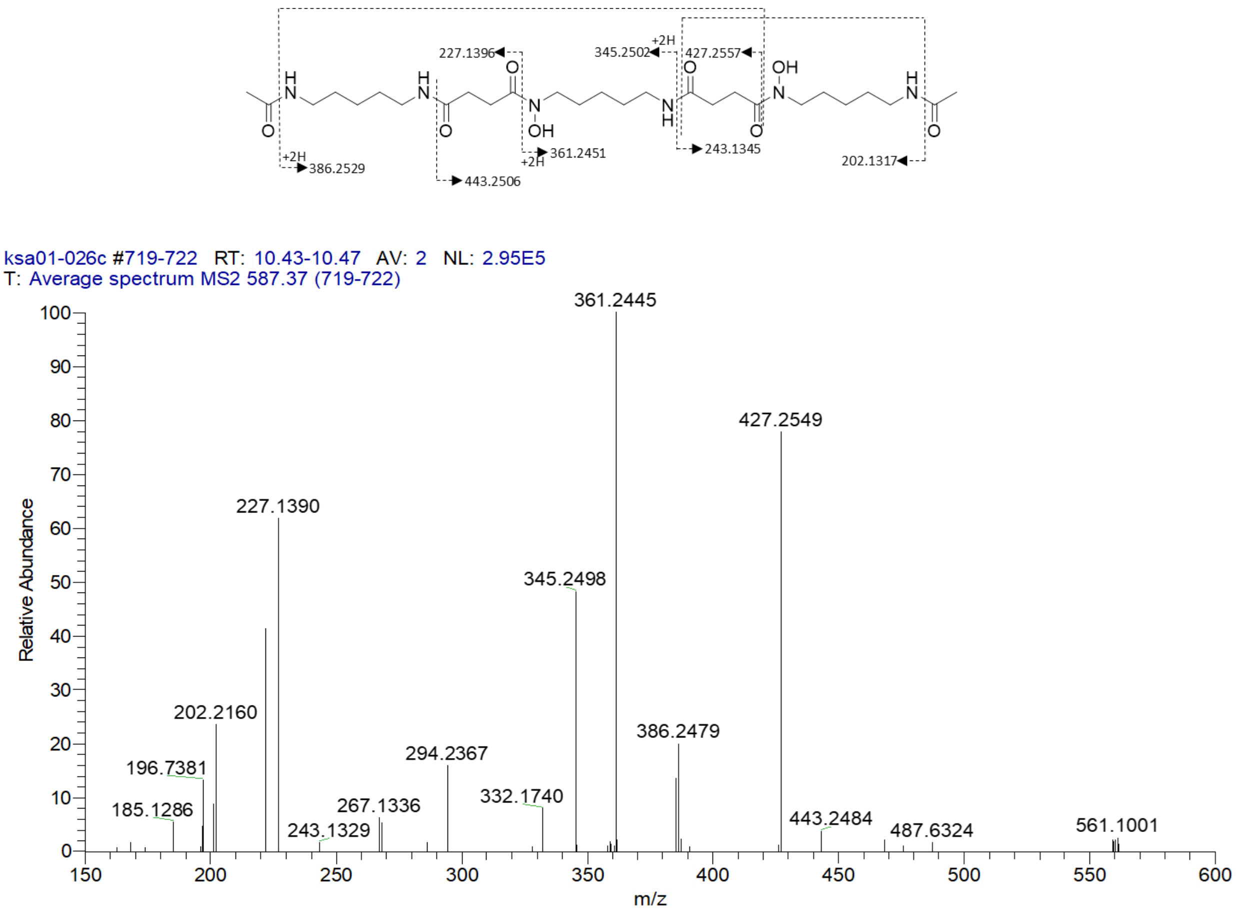

Figure S34. MS/MS spectrum of desoxy-desferrioxamine D_1 **2** (587.3754 $[M + H]^+$) with annotation in the structure.

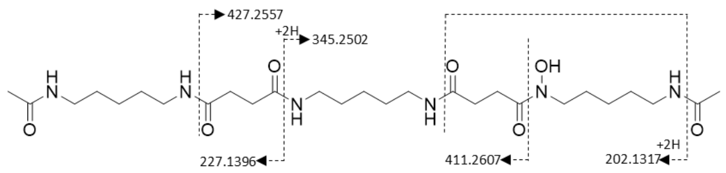

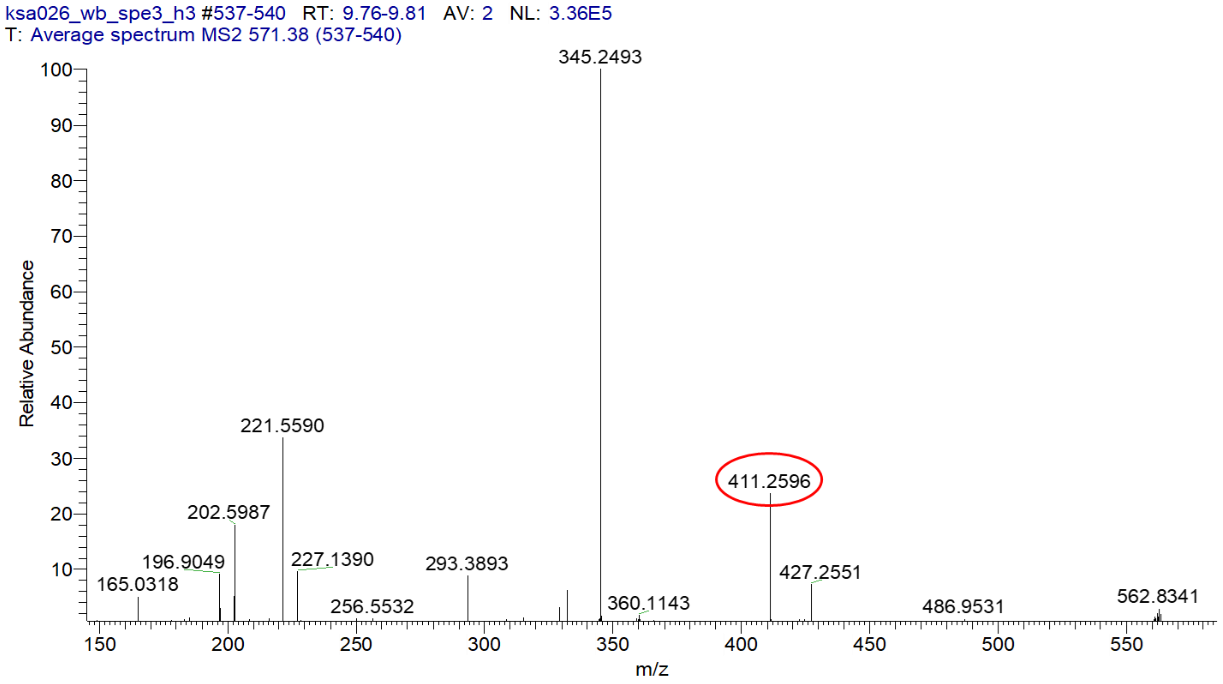

Figure S35. MS/MS spectrum of *di*desoxy-desferrioxamine D_1 **8** (571.3807 $[M + H]^+$) with annotation in the structure.

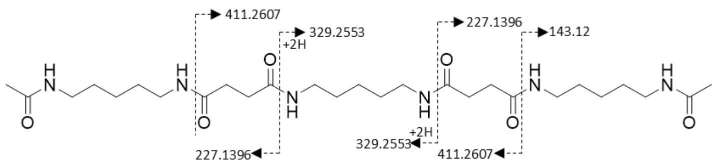

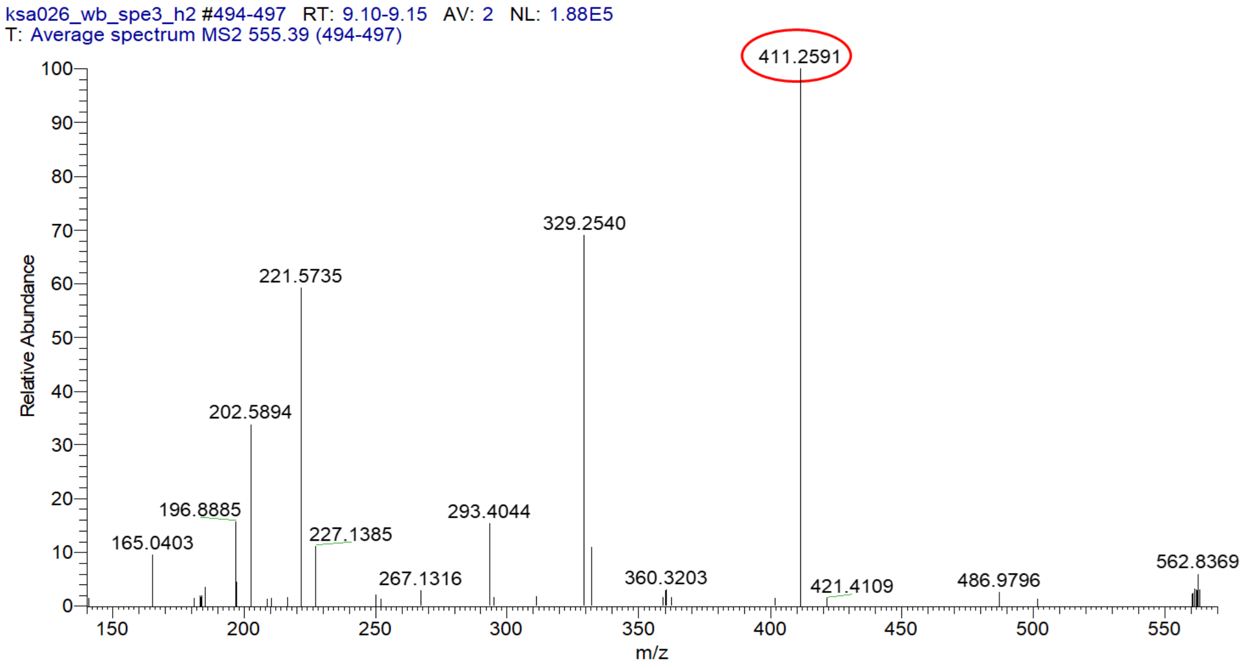

Figure S36. MS/MS spectrum of speibonoxamine **1** (555.3857 [M + H]$^{+}$) with annotation in the structure.

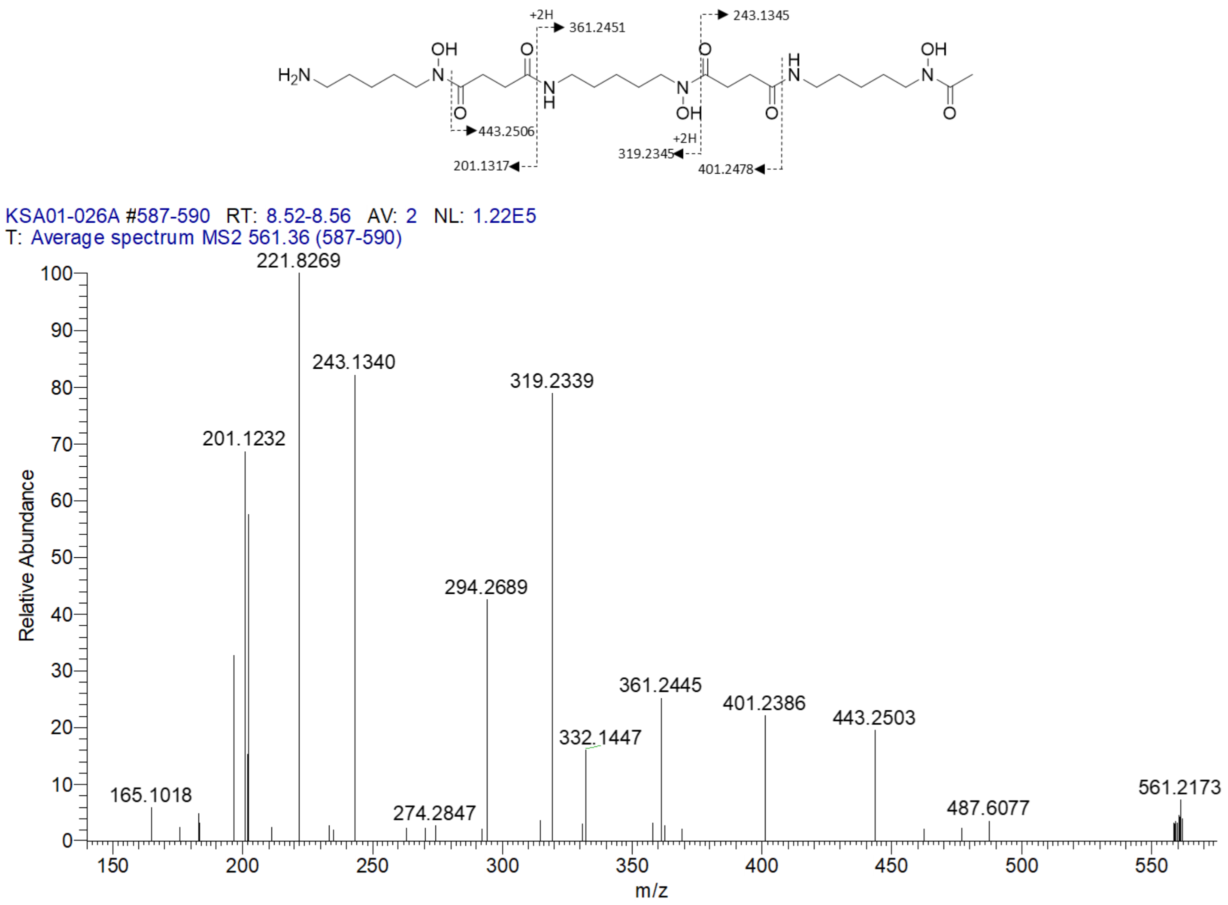

Figure S37. MS/MS spectrum of desferrioxamine B **4** (561.3606 [M + H]$^+$) with annotation in the structure.

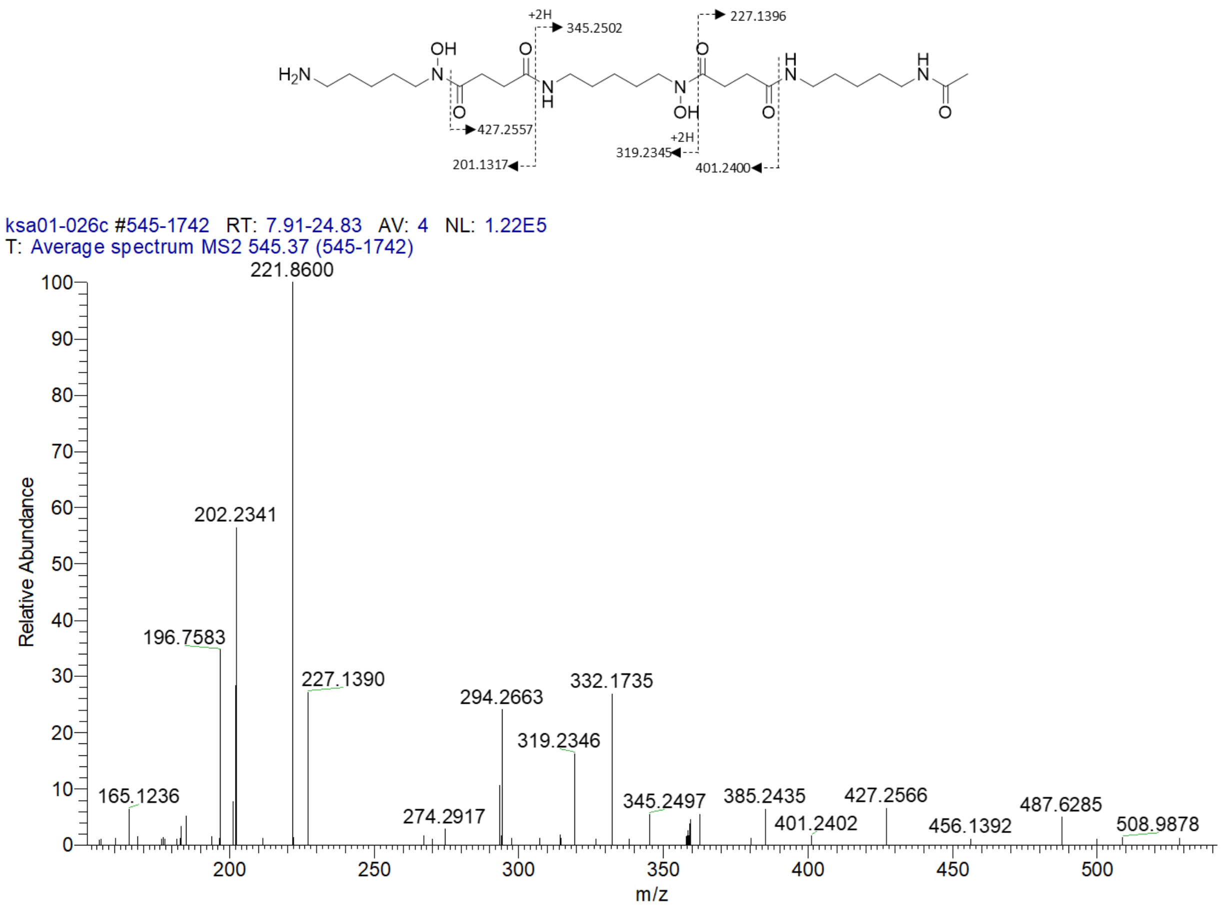

Figure S38. MS/MS spectrum desoxy-desferrioxamine B **9** (545.3654 [M + H]$^+$) with annotation in the structure.

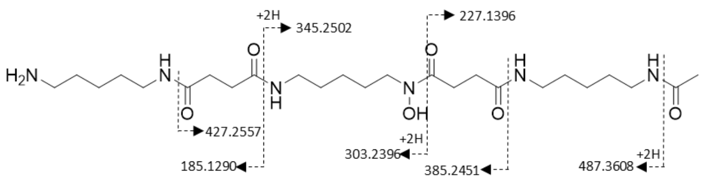

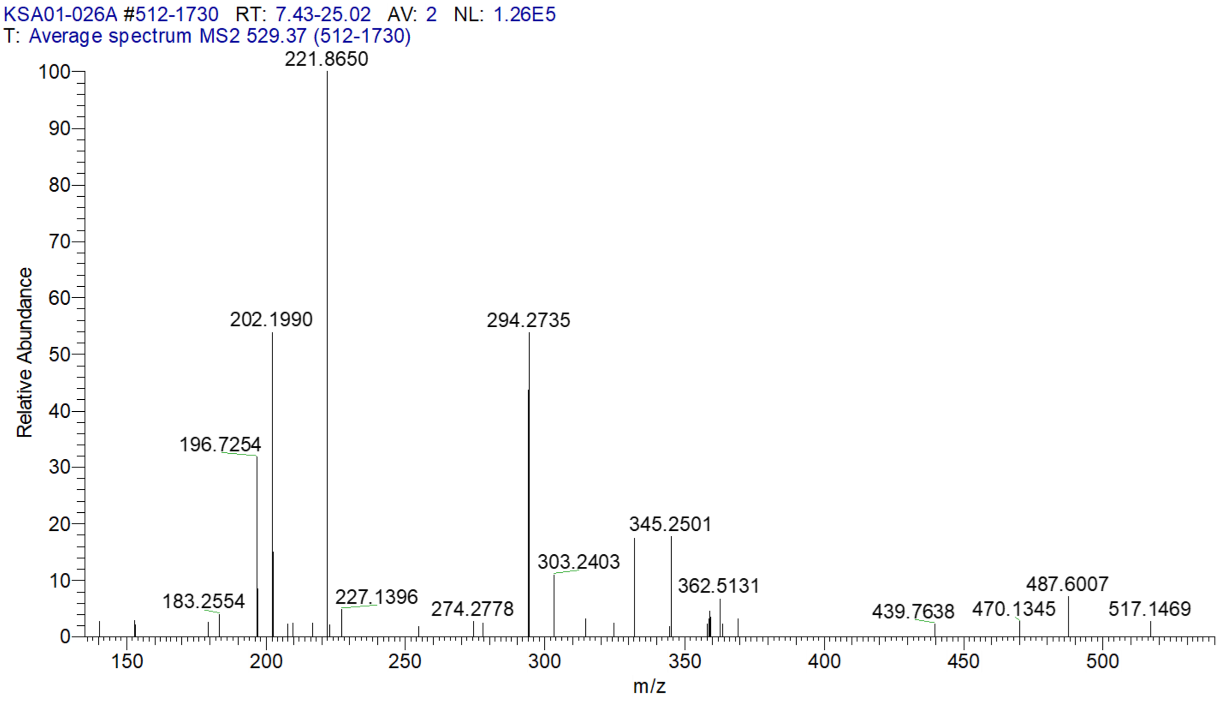

Figure S39. MS/MS spectrum *di*desoxy-desferrioxamine B **10** (529.3702 [M + H]$^+$) with annotation in the structure.

Table S1. Compounds isolated and tentatively identified in the molecular cluster and the MS chromatogram of the fermentation broth of *K. speibonae* strain SK5 with their corresponding masses, molecular formulae (MF), double bond equivalent (DBE), retention time (Rt) and mass error (ID (Δ ppm))

Compound	m/z ($[M+H]^+$)	m/z ($[M+H]^+$) Calculated	MF	DBE	Rt	ID Δ ppm
Speibonoxamine **1**	555.3857	555.3870	$C_{27}H_{50}N_6O_6$	6	9.13	1.004
Desoxy-desferrioxamine D_1 **2**	587.3754	587.3768	$C_{27}H_{50}N_6O_8$	6	10.41	-2.092
Desferrioxamine D_1 **3**	603.3712	603.3718	$C_{27}H_{50}N_6O_9$	6	11.03	-0.685
Desferrioxamine B **4**	561.3606	561.3612	$C_{25}H_{48}N_6O_8$	5	8.63	-1.049
Desoxynocardamine **5**	585.3606	585.3612	$C_{27}H_{48}N_6O_8$	7	10.40	-0.391
Desferrioxamine E **6**	601.3559	601.3561	$C_{27}H_{48}N_6O_9$	7	11.11	0.161
hexahydro-3-[(4-hydroxyphenyl)methyl]-pyrrolo[1,2-a]pyrazine-1,4-dione **7**	261.1235	261.1239	$C_{14}H_{16}N_2O_3$	8	5.29	1.459
*Di*desoxy-desferrioxamine D_1 **8**	571.3807	571.3819	$C_{27}H_{50}N_6O_7$	6	9.87	-1.005
Desoxy-desferrioxamine B **9**	545.3654	545.3663	$C_{25}H_{48}N_6O_7$	5	7.94	-0.668
*Di*desoxy-desferrioxamine B **10**	529.3702	529.3714	$C_{25}H_{48}N_6O_6$	5	7.40	-1.076
Desferri-ferrioxamine H **11**	461.2606	461.2611	$C_{20}H_{36}N_4O_8$	5	7.99	-0.608
Ferrioxamine B **12**	614.2720	614.2727	$C_{25}H_{45}FeN_6O_8$	5	4.89	0.370
Ferrioxamine E **13**	654.2670	654.2676	$C_{27}H_{45}FeN_6O_9$	7	6.64	0.330
Ferrioxamine D_1 **14**	656.2820	656.2832	$C_{27}H_{47}FeN_6O_9$	6	7.50	0.710
Arthrobactin **15**	477.2555	477.2561	$C_{20}H_{36}N_4O_9$		8.63	-0.849
hexahydro-3-(phenylmethyl)-pyrrolo[1,2-a]pyrazine-1,4-dione **16**	245.1288	245.1290	$C_{14}H_{16}N_2O_2$	8	9.11	0.961
